# Supplementary material for: Role of cash transfers in mitigating food insecurity in India during the COVID-19 pandemic: a longitudinal study in the Bihar state
Source: BMJ Open. 2022 Jun 27;12(6):e060624. doi: 10.1136/bmjopen-2021-060624 (PMC9237649; doi:10.1136/bmjopen-2021-060624)
Supplement: Supplementary data [file bmjopen-2021-060624supp001.pdf]

Supplementary Table 1: Household characteristics affecting participation in the Survey 2[1] (N=1713)

Whether the household participated in Survey 2 or not	Odds Ratio	95% Confidence Interval
Food Insecure: No[2]		
Yes	1.05	0.81, 1.35
PDS Beneficiary: Yes[2]		
No	1.02	0.84, 1.24
Land owned (acres)	0.96	0.91, 1.01
Household size	0.98	0.94, 1.02
Household head education status: literate[2]		
Illiterate	1.54	1.26, 1.90
Age (years)	1.00	1.00, 1.00
Positive or no income shock[2,3]		
Negative Income shock	1.09	0.87, 1.38
Employment category: Casual labour[2]		
Regular Salaried	1.01	0.75, 1.36
Self Employed in Agriculture	1.02	0.79, 1.30
Self Employed in Non-agriculture	0.90	0.64, 1.27
MPCE categories[4]: Quintile 1[2]		
Quintile 2	0.82	0.60, 1.11
Quintile 3	0.81	0.59, 1.10
Quintile 4	0.75	0.55, 1.02
Quintile 5	0.86	0.62, 1.18

[1]*Analysis using logistic regression.*
[2]*Reference category.*
[3]*No or positive economic shocks includes found a job, hike in salary and received food and money as gift whereas negative economic shock includes business closures, mass layoffs, price increase of commodities, job loss, wage cuts, loss of remittances, low rate for produce to be sold at market price, indebtedness, and crop failure.*
[4]*Monthly per capita expenditure quintiles as a proxy for income quintile.*
