# Supplementary material for: Role of cash transfers in mitigating food insecurity in India during the COVID-19 pandemic: a longitudinal study in the Bihar state
Source: BMJ Open. 2022 Jun 27;12(6):e060624. doi: 10.1136/bmjopen-2021-060624 (PMC9237649; doi:10.1136/bmjopen-2021-060624)
Supplement: Supplementary data [file bmjopen-2021-060624supp002.pdf]

Supplementary Table 2: Household characteristics by cash benefit status in the post lockdown period (N=859)

		Households who did not receive cash benefits (N=497)	Households who received cash benefits (N=362)	p-value
Age(years)		40 [30,58.5]	42 [30,55]	0.79
Household size		7 [5,9]	7 [6,9]	0.078
Land owned (hectares)		0.63 [0.13,2.58]	0.25 [0.08,1.95]	0.016
Household head education	Illiterate	334 (67.2%)	240 (66.3%)	0.78
	Literate	163 (32.8%)	122 (33.7%)	
Household head gender	Male	448 (90.1%)	315 (87.0%)	0.15
	Female	49 (9.9%)	47 (13.0%)	
PDS Beneficiary	Yes	231 (46.5%)	209 (57.7%)	0.001
	No	266 (53.5%)	153 (42.3%)	
Caste category	Forward class	64 (12.9%)	41 (11.3%)	0.79
	Other backward classes	289 (58.1%)	215 (59.4%)	
	Dalit & mahadalits	144 (29.0%)	106 (29.3%)	
Employment category	Casual labourer	117 (23.5%)	91 (25.1%)	0.93
	Regular Salaried	95 (19.1%)	64 (17.7%)	

	Self Employed in Agriculture	229 (46.1%)	166 (45.9%)	
	Self Employed in Non-agriculture	56 (11.3%)	41 (11.3%)	
Household wealth status	Quintile 1	88 (17.7%)	67 (18.5%)	0.66
	Quintile 2	97 (19.5%)	76 (21.0%)	
	Quintile 3	97 (19.5%)	77 (21.3%)	
	Quintile 4	105 (21.1%)	77 (21.3%)	
	Quintile 5	110 (22.1%)	65 (18.0%)	

**Numbers are presented as median (Inter-Quartile range) for continuous measures, and frequency (%) for categorical measures*
