# Supplementary material for: Role of cash transfers in mitigating food insecurity in India during the COVID-19 pandemic: a longitudinal study in the Bihar state
Source: BMJ Open. 2022 Jun 27;12(6):e060624. doi: 10.1136/bmjopen-2021-060624 (PMC9237649; doi:10.1136/bmjopen-2021-060624)
Supplement: Author's manuscript [file bmjopen-2021-060624.draft_revisions.pdf]

BMJ Open is committed to open peer review. As part of this commitment we make the peer review history of every article we publish publicly available.

When an article is published we post the peer reviewers' comments and the authors' responses online. We also post the versions of the paper that were used during peer review. These are the versions that the peer review comments apply to.

The versions of the paper that follow are the versions that were submitted during the peer review process. They are not the versions of record or the final published versions. They should not be cited or distributed as the published version of this manuscript.

BMJ Open is an open access journal and the full, final, typeset and author-corrected version of record of the manuscript is available on our site with no access controls, subscription charges or pay-per-view fees (http://bmjopen.bmj.com).

If you have any questions on BMJ Open's open peer review process please email info.bmjopen@bmj.com

BMJ Open

The role of cash transfers in mitigating food insecurity in India during the COVID-19 pandemic: the case of Bihar state

Journal:	*BMJ Open*
Manuscript ID	bmjopen-2021-060624
Article Type:	Original research
Date Submitted by the Author:	04-Jan-2022
Complete List of Authors:	Makkar, Sanchit; St John's Research Institute, Division of Nutrition<br>Manivannan, Jawahar ; St John's Research Institute, Division of Epidemiology and Biostatistics<br>Swaminathan, Sumathi; St John's Research Institute, Division of Nutrition<br>Travasso, Sandra ; St John's Research Institute, Division of Epidemiology and Biostatistics<br>John, Anjaly ; St John's Research Institute, Division of Epidemiology and Biostatistics<br>Webb, Patrick ; Tufts University Friedman School of Nutrition Science and Policy<br>Kurpad, Anura; St John's Medical College, Department of Physiology<br>Thomas, Tinku; St John's Medical College, Department of Biostatistics
Keywords:	COVID-19, STATISTICS & RESEARCH METHODS, PUBLIC HEALTH

SCHOLARONE™
Manuscripts

I, the Submitting Author has the right to grant and does grant on behalf of all authors of the Work (as defined in the below author licence), an exclusive licence and/or a non-exclusive licence for contributions from authors who are: i) UK Crown employees; ii) where BMJ has agreed a CC-BY licence shall apply, and/or iii) in accordance with the terms applicable for US Federal Government officers or employees acting as part of their official duties; on a worldwide, perpetual, irrevocable, royalty-free basis to BMJ Publishing Group Ltd ("BMJ") its licensees and where the relevant Journal is co-owned by BMJ to the co-owners of the Journal, to publish the Work in this journal and any other BMJ products and to exploit all rights, as set out in our licence.

The Submitting Author accepts and understands that any supply made under these terms is made by BMJ to the Submitting Author unless you are acting as an employee on behalf of your employer or a postgraduate student of an affiliated institution which is paying any applicable article publishing charge ("APC") for Open Access articles. Where the Submitting Author wishes to make the Work available on an Open Access basis (and intends to pay the relevant APC), the terms of reuse of such Open Access shall be governed by a Creative Commons licence – details of these licences and which Creative Commons licence will apply to this Work are set out in our licence referred to above.

Other than as permitted in any relevant BMJ Author's Self Archiving Policies, I confirm this Work has not been accepted for publication elsewhere, is not being considered for publication elsewhere and does not duplicate material already published. I confirm all authors consent to publication of this Work and authorise the granting of this licence.

For peer review only

The role of cash transfers in mitigating food insecurity in India during the COVID-19 pandemic: the case of Bihar state

Sanchit Makkar[1], Jawahar Ramasamy Manivannan[2], Sumathi Swaminathan[1], Sandra M. Travasso[2], Anjaly Teresa John[2], Patrick Webb[3], Anura V Kurpad[4], Tinku Thomas[5]

[1] Division of Nutrition, St. John's Research Institute, Bengaluru, India

[2] Division of Epidemiology and Biostatistics, St. John's Research Institute, Bengaluru, India

[3] Friedman School of Nutrition Science and Policy, Tufts University, Boston, U.S.A

[4] Department of Physiology, St. John's Medical College, Bengaluru, India

[5] Department of Biostatistics, St. John's Medical College, Bengaluru, India

Corresponding Author:

Dr. Tinku Thomas

Department of Biostatistics,

St. John's Medical College, Bengaluru, India

E-mail: tinku.sarah@sjri.res.in

Word count: 3997

For peer review only

Abstract

Objective: There are scant empirical data on the impacts of the Coronavirus (COVID-19) pandemic on food security across the globe. India is no exception, with insights into the impacts of lockdown on food insecurity now emerging. We contribute to the empirical evidence on the prevalence of food insecurity in Bihar state before and after lock-down, and whether the government's policy of cash transfer moderated negative effects of food insecurity or not.

Design: This was a longitudinal study.

Settings: The study was conducted in Gaya and Nalanda district of Bihar state in India from December 2019 to September 2020.

Participants: A total of 1797 households were surveyed in Survey 1 and about 52% (n=939) were followed up in survey 2. Valid data for 859 households were considered for the analysis.

Main outcome measures: Using the Food Insecurity Experience Scale (FIES), household conditions were compared pre- and post-lockdown. The effect of cash transfers was examined in a quasi-experimental method using a longitudinal study design. Logistic regression and propensity score adjusted analyses were used to identify factors associated with food insecurity.

Results: Household food insecurity worsened considerably during lockdown, rising from 20% (95% CI:17.4-22.8) to 47% (95% CI: 43.8-50.4) at the sample mean. Households experiencing negative income shocks were more likely to have been food insecure before the lockdown (Odds Ratio=6.4, 95% CI:4.9-8.3). However, households that received cash transfers had lower odds of being food insecure once the lockdown was lifted (Odds Ratio=0.75, 95% CI:0.56-0.99).

For peer review only

Conclusion: These findings provide evidence on how the swift economic response to the pandemic crises using targeted income transfers were relatively successful in mitigating potentially deep impacts of food insecurity.

Keywords:

Cash transfers, COVID-19, Food insecurity, India

For peer review only

For peer review only - http://bmjopen.bmj.com/site/about/guidelines.xhtml

Strengths and limitations of this study

- We studied the effect of COVID -19 pandemic on Household Food Insecurity and role of cash transfers in mitigating this effect if any in the Bihar state of India.
- We used Rasch modelling to statistically validate the food insecurity information collected through longitudinal survey.
- Role of cash transfers were analyzed in a longitudinal study design using quasi-experimental approach after addressing for endogeneity.
- The follow up survey conducted telephonically led to drop out of some households and potentially affected the way enumerators collected information.

For peer review only

1. Introduction

The COVID-19 pandemic is known to have had serious negative effects across the world, with particularly concerning implications for poverty, malnutrition, and food insecurity in low- and middle-income countries (LMICs). However, empirical evidence of such impacts remain scarce [1]. Food insecurity is defined as "when a person lacks regular access to enough safe and nutritious food for normal growth and development and an active and healthy life. This may be due to unavailability of food and/or lack of resources to obtain food. Food insecurity can be experienced at different levels of severity" [2]. It is estimated that in South and Southeast Asia, around 33 million people may have been pushed into a state of acute food insecurity since February 2020 [3], and that between 88 and 115 million people globally were pushed into extreme poverty during 2020 [4].

While governments scrambled to curb the spread of the disease, one of the most common policy measures was to prevent the movement and congregation of individuals by imposing lockdowns that were intended to isolate vulnerable people and prevent transmission. An unfortunate (but foreseen) side-effect of lockdown was increased poverty and constrained access to food due to an inability to leave the home, and because of loss of jobs and income. In India, a rigorous lockdown imposed on 23rd March 2020 brought most economic activities to a halt. The abrupt lockdown caused disruptions to food supplies, restricted labor availability and severely cut incomes, resulting in an economic downturn that affected most of India's 1.3 billion population [5]. A survey by the Centre for Monitoring Indian Economy (CMIE) showed a steep rise in unemployment rate across India from 8.4% in March 2020 to 23.5% in April 2020, with 75% (91 million) attributed to job loss among small traders and casual labor [6,7]. During the period of lockdown, the prices for non-cereal nutrient-dense foods (pulses, vegetables and eggs) rose faster and higher than the price of cereal foods like wheat and rice [8]. In addition, the government's long-standing supplementary feeding programs, specifically

For peer review only - http://bmjopen.bmj.com/site/about/guidelines.xhtml

the Integrated Child Development Services and the Mid-day Meal Program, were suspended, which negatively impacted food consumption of entitled children and women. In an attempt to mitigate the expected adverse effects of the lockdown, the government implemented an urgent relief package valued at INR 1.7 lakh crore (~US$22.6 billion), including both cash and food support [9]. This paper focuses on the cash transfers and its effect on food insecurity.

Cash transfers can play a significant role in diversifying the diet and improving household food consumption [10,11], because it can smooth the variability of food consumption by stabilizing income, thereby protecting food intake at household level [12]. However, the impact of cash transfer initiative specific to Covid-19 has not been studied so far. Our specific objective was to document the state of food insecurity in two districts of Bihar and the role (if any) of cash transfers in mitigating negative impacts on food insecurity at the household level.

2. Methodology

Sources of Data

We conducted a longitudinal study in Gaya and Nalanda districts in Bihar. A door-to-door survey was conducted from July 2019 through September 2019, and a follow-up (denoted as Survey 1) survey was undertaken from December 2019 through February 2020 (n=1732 households). Of 1797 households contacted, valid data were available for 1732. The original intent was to examine linkages between agriculture and nutrition in rural areas, with a focus on five nutrient-dense foods: namely, pulses (red lentils), milk, green leafy vegetables, eggs and poultry. A total of 142 villages in Gaya district and 134 in Nalanda district were sampled through multistage cluster sampling, and households were chosen by the random walk method (10 households per village). To account for heterogeneity in distances to district market centers, the villages were sampled from 0-5 km, 6-15 km, 16-30 km, and >30 km distance bands from

For peer review only - http://bmjopen.bmj.com/site/about/guidelines.xhtml

the district headquarters. Within each village, households involved in the production of the nutrient-dense foods, landholding households (regardless of their output), and landless households were included in the sampling frame. Data on household consumption of food in the previous month was collected from all households, and information related to food production was collected from those households involved in the production of nutrient-dense foods.

When lockdown was eased, a telephonic survey (Survey 2) was conducted in the same households during August and September 2020. Using the contact numbers (1797 households) provided during Survey 1, all respondents were contacted telephonically. After multiple attempts, response rate was 52.3% (n=939) with >50% response rate in each district. The aim of the telephonic survey was to identify the effects of the pandemic on food production and consumption in the context of restrictions. All interviews were audio recorded, as well as documented in interviewer notes. The head of the household or his/her spouse were the respondents and the interviews were in Hindi.

The survey instrument included questions on the effects of COVID-19 on participant income/livelihoods, effects on food production and sale (among food producing households), support received in cash and in-kind from the government, NGOs and others during the period, and effects on food purchase and consumption (among all respondents). The widely validated Food Insecurity Experience Scale (FIES) that determines food access at different levels of severity was also administered. Complete FIES data were available from 1713 households in Survey 1 and 859 in Survey 2 were considered for the analysis. **Figure 1** provides the framework, laying out the data sources used, and methodology adopted for the study.

For peer review only - http://bmjopen.bmj.com/site/about/guidelines.xhtml

The Food Insecurity Experience Scale (FIES)

The household version of the Food Insecurity Experience Scale (FIES) survey was administered to all sampled households in survey 1 and 2. The FIES has a set of eight questions with dichotomous responses (Yes or No). Specifically, to determine access to food over the prior month, questions whether the household; a) worried about not having enough food, b) was unable to eat healthy and nutritious food, c) ate only few kinds of foods, d) skipped a meal, e) ate less, f) ran out of food, g) was hungry but did not eat and h) went without eating for a whole day [13] were asked. A Rasch model was used to assess the appropriateness of the FIES data collected in the context of the current study, as recommended by FAO [13]. The model was used to statistically validate the quality of the data based on assumptions such as (1) more severe items are less frequently affirmed and (2) household that affirm a particular item on the scale are likely to affirm lesser severe items on the scale. Although these are not necessary conditions, testing of these served as a validation for the item scoring. The Rasch model is a single parameter logistic item response theory model and was used to validate FIES based on these assumptions, building on the precursor Household Food Insecurity and Access Scale (HFIAS) [14]. The domains identified as significant components of food insecurity are comparable across ethnographic groups [15] and therefore establishes the appropriateness of the tool for the setting.

The four different types of results generated from the Rasch model, which are used to check the assumptions made, are fit statistics which are infit and outfit, residual correlation matrix and Rasch reliability criteria. Infit statistic measures the discriminatory power of an item i.e., items that did not perform well within the given data. The outfit statistic is like infit, but it is sensitive to responses which are extremely unexpected or outliers in the response pattern. To validate the data, a set of criteria for these measures were followed [13,16]. The adequate fit value for both fit statistics had to lie between 0.7 to 1.3. If an outfit value was >2,

For peer review only - http://bmjopen.bmj.com/site/about/guidelines.xhtml

it was considered high. However, if infit statistics were well within range then high outfit values were not considered for eliminating the items. The residual correlation matrix for each pair of items indicates pairs that are redundant and an absolute value > 0.4 was considered high indicating multidimensionality between pair of items. The last assumption (i.e., Rasch Reliability) accounts for the total variance explained by the model. For 8 items FIES scale, a value greater than 0.7 is considered reliable and adequate.

Following statistical validation, the households having a raw score ≤ 3 [16] were identified to be 'food secure'. This category would also include mild food insecure households. The households were categorized to be food insecure for a raw score >3. This binary classification of food insecurity status was used as the outcome variable in the entire analysis.

Statistical Methods

Household factors associated with food insecurity were identified through logistic regressions using the Survey 1 data on 1713 households. The effect of cash transfers on food insecurity was examined in Survey 2 (n=859) data using logistic regression after adjusting for all household factors identified to be associated with food insecurity in Survey 1 analysis. However, cash transfers were based on an eligibility criterion, which implies a non-randomization in sample selection. Therefore, it may cause endogeneity that could arise due to household characteristics being conducive to receiving cash benefit and hence a potential selection bias leading to biased estimate and standard errors with narrower confidence interval.

Further, as a sensitivity analysis to account for potential endogeneity, we computed the propensity score for receipt of cash benefit considering household characteristics that were either associated with cash benefit or with household food insecurity. The variables considered for the computation of the propensity score were: having a ration card or beneficiary of Public Distribution System (PDS) that provides food and non-food items through fair price shops at a

For peer review only - http://bmjopen.bmj.com/site/about/guidelines.xhtml

subsidized rates (yes vs no), household size, land owned (acres), education status (literate vs illiterate) and age of the household head, type of economic activity household is involved (casual labor, regular salaried, self-employed in agriculture and non-agriculture) and wealth quintiles from monthly per capita expenditure.

The difference in change in the odds of being food insecure between Survey 1 to Survey 2 (for households that received and did not receive cash benefits) was examined as an interaction effect between time (pre-lockdown survey and post-lockdown survey) and receipt of cash transfer, in a random effects model with logit link function, using the propensity score as a covariate. The following generalized estimating linear model was used,

$$\text{Logit}(p_{ij}) = \beta_0 + \beta_1 C_{ij} + \beta_2 T_{ij} + \beta_3 (C_{ij}*T_{ij}) + B_4 P_{ij} + \beta_5 X_{ij} + \epsilon_{ij}$$

Where p_{ij} is the probability of household food insecurity for household *i* in time *j* where *j* is 0 (Survey 1) and 1 (Survey 2); C_{ij} is an indicator variable for the household *i* having received cash benefit in time *j*; T_{ij} is the time dummy being equal to 0 for pre lock down period and 1 for post lock down period for household *i*; β_1 is the population average difference in log odds of food insecurity by cash benefit and β_2 is the population average difference in log odds of food insecurity between the two rounds of survey; $C_{ij}*T_{ij}$ is the interaction between the time and receipt of cash transfer. Statistical significance of interaction effect was considered at $p<0.1$. The coefficient β_3 gives the estimated effect of cash transfer on food insecurity over time; P_{ij} is the propensity score for each household; X_{ij} is a matrix containing household characteristics in time j and ϵ_{ij} is the error term.

All results are expressed as odds ratio (95% confidence interval). All analysis were performed using STATA version 16.1.

Patient and Public Involvement

No Patient involved

For peer review only - http://bmjopen.bmj.com/site/about/guidelines.xhtml

3. Results

Socioeconomic characteristics of population before lockdown

Data from 1713 households were included for this analysis (**Table 1**). The median household size was 6 [IQR 5,8] and 11% were women-headed households. Overall, only 39% of household heads were literate, of whom 80% were male. The median area of land owned by households was 0.09 acres. About 51% of the households reported having a ration card, implying that they were beneficiaries of safety net program, the Public Distribution System (PDS). About 25% of all households reported that they were engaged in casual labor, while about 18% had regular salaries, and 56% were self-employed in agriculture and non-agriculture economic activity.

Statistical validation of food insecurity using Rasch modelling

The infit statistics were examined separately in the subgroup of households who participated in both surveys, and therefore made up the longitudinal panel (N=859). The infit statistics were adequately within the range i.e., 0.7 to 1.3, suggesting that the assumption of equal discrimination is met, and all the items are related to the latent trait. However, items such as 'worried' and 'healthy' had the outfit statistic > 2 in both surveys. Since the infit statistics were within range, these items were not dropped based on the outfit measure. Hence, all the 8 items were retained to calculate the prevalence of food insecurity. The overall fit of the model i.e., Rasch reliability criteria (0.74 and 0.71 for Survey 1 and Survey 2 respectively) was greater than 0.7, suggesting a reasonably good fit. The residual correlations between pairs of items were all <0.4 implying no conditional dependence between the pairs of items. In addition, we checked these assumptions for the entire Survey 1 sample (N=1713) and similar results were obtained. The raw scores from all 8 items were used to classify households as food insecure (>3) or not in the two surveys.

For peer review only - http://bmjopen.bmj.com/site/about/guidelines.xhtml

Prevalence of food insecurity pre- and post-lockdown

The prevalence of food insecurity before lockdown was similar in the subsample that participated in Survey 2 (20.1%) as in the entire sample of Survey 1 (21.5%) (**Table 2**). This implies that the Survey 2 sample (n=859) was representative of the population covered in Survey 1 (**Table 2**). The prevalence of food insecurity in the sub-sample from Survey 1 increased significantly from 20.1% pre-lockdown to 47.1% post-lockdown (P < 0.001), underlining the immediate impact of COVID-19 on the food basket of households.

Factors affecting food insecurity before lockdown

In the pre-lockdown survey of 1713 households (**Table 3**), the literacy of the household head was significantly associated with food insecurity (OR: 1.41, 95%CI: 1.08, 1.83). Households that had experienced a recent negative economic shock, such as job loss or fall in income, were 6.4 times more likely to suffer food insecurity compared to households that reported either no negative or positive economic shocks, including the finding of a new job (OR: 6.41, 95%CI: 4.97, 8.27). PDS beneficiary status was not associated with food insecurity status. People engaged in a regular salaried profession or self-employment in agriculture had lower odds of experiencing food insecurity (OR: 0.65, 95%CI: 0.45, 0.94 and OR: 0.69, 95%CI: 0.5, 0.95, respectively) compared to casual labourers. Belonging to a higher income class i.e., top 40% (quintile 4 and quintile 5), had lower odds of being food insecure as compared to bottom 20% income class (OR: 0.63, 95%CI: 0.42, 0.94 and OR: 0.66, 95%CI: 0.43, 1.00, respectively).

Factors affecting food insecurity over time, including cash transfers

In the 859 households surveyed in the post-lockdown period, about 42% households had received a cash transfer from the government. The total amount received during the 3 lockdown months (April to June 2020) ranged from INR 200 to INR 7500, with households

For peer review only - http://bmjopen.bmj.com/site/about/guidelines.xhtml

reporting the receipt of a median INR 1500 [IQR: INR 1000, INR 1600]. In addition, 55% of the households also received food as well as other in-kind benefits from various Non-Governmental Organizations (NGOs), and 28% received both cash and in-kind benefits.

Households that received cash transfers had a lower proportion of food insecurity (43.6%) compared to those who did not (49.7%). Among factors that influenced food security in the post lockdown period (**Table 4**), households that received the transfers were 25% less likely to be food insecure compared to those that did not (OR: 0.75, 95%CI: 0.56, 0.99). Other factors such as type of employment, wealth quintiles were associated with food insecurity in the same manner as the pre-lockdown period. The lockdown related factors reported by households that could have contributed to food insecurity were restrictions in agricultural activities (42.3%), increased purchase prices (22%-53% depending on the type of food) and negative effect on livelihood (79.3%).

To arrive at better estimates of the effects of cash transfer on food insecurity, longitudinal data (pre and post lockdown surveys) of 859 household were considered. The households that received cash transfer were compared with those who did not. The proportion of PDS beneficiary households were 58% in the group that received the cash transfer (**Supplementary Table 1**) compared to 47% in the group that did not receive this. All other characteristics were comparable between the groups. Therefore, cash benefits could be conditional on eligibility criteria which we could not assess directly. Hence, we also corrected for endogeneity arising due to selection bias by propensity scores adjusted analysis (**Table 5**). The interaction effect of time (pre- vs post-lockdown) and receipt of cash transfer (received cash benefits or not received) was examined to quantify the differential effect of cash benefit on household food insecurity change due to the lockdown. Overall, there was a significant increase in household food insecurity in the post-lockdown survey compared to pre-lockdown (OR: 4.5, 95%CI: 3.4, 5.93). The interaction of time and cash benefit receipt was significant

For peer review only - http://bmjopen.bmj.com/site/about/guidelines.xhtml

(OR: 0.65, 95%CI: 0.42, 1.01, p=0.06) such the odds of becoming food insecure in the lockdown period compared to pre-COVID, by the group that received cash benefits in the post lockdown period was only 0.65 times compared to the households that did not receive cash benefits. This clearly demonstrated a beneficial effect of cash transfer on food insecurity even after adjusting for differences in familial characteristics between the households that received and did not receive cash benefits.

4. Discussion

This study contributes evidence on the impact of the COVID pandemic related lockdown on food security in Bihar state, in two ways. First, it examines the status of food insecurity in the pre- and post- lockdown periods. Second, it examines the impact of cash transfers during this period on post- lockdown food insecurity. Our longitudinal survey findings suggest that there was a significant increase in the prevalence of food insecurity immediately post-lockdown, such that this increased steeply, from 20% (pre- lockdown) to 47% (post-lockdown). We also found that the social safety net programs such as cash transfers introduced under the Pradhan Mantri Garib Kalyan Yojana (PM-GKY), had the potential to address this stressful situation, such that the odds of being food insecure in a given household sample were much lower in comparison to households that did not receive the cash transfer. The transfers may have acted as an added income for the households, such that their purchasing power to spend on essential items did not reduce. The paper also examines how the cash transfer mechanism was beneficial to some degree to tackle this crisis.

Intervention strategies focused on reducing poverty and food insecurity in the context of COVID-19 have been widely implemented across LMICs, but not really assessed in terms of their impacts [17,18]. For example, India announced a relief package in March 2020 as an immediate response to the economic crisis associated with its policy decision to lockdown.

For peer review only - http://bmjopen.bmj.com/site/about/guidelines.xhtml

Although these existing schemes were important, they did not ensure protection of the various dimensions that affect livelihoods and consequently, food affordability.

The supplementary nutrition such as Integrated Child Development Services, Mid-day meal programs etc. were disrupted during the lockdown period and these programs go a long way in providing quality nutrition to the beneficiary population. This study shows that a cash transfer of approximately INR 500/month was helpful in mitigating the negative effect of the lockdown on food insecurity and was helpful towards meeting the energy requirement of the recipient household. However, the sufficiency of this amount to meet the requirements for dietary diversity of the beneficiary households is still questionable. Some evidence suggests that cash transfers increase local food prices, steepened the price slope and potentially worsened food security for those who were ineligible for transfers [19–21] but this was not investigated in the current study. Our research was limited to understanding the immediate impact of cash transfers on household food insecurity. In the long run, even after the lifting of the Covid-19 lockdown, the bigger problem is the need to holistically meet the nutritional requirements of the population, rather than simply their energy requirements or averting hunger. This is because the revival of the economic status of the population is likely to take longer, especially with Covid-19 related restrictions still persisting, and with repeated waves of infection.

The effect of the pandemic on food insecurity found in this study is externally valid in India, as a study in Uttar Pradesh state of India showed findings consistent with the present Bihar study, where household food insecurity increased from 21% in December 2019 to 80% August 2020 and children in these households were less likely to consume a diverse diet [22]. As far as food security among the Indian farmers is concerned, a study on tomato and wheat producers showed that the latter were relatively less affected in terms of decline in income due to fixed market prices and hence, were less affected by food insecurity in comparison to the

For peer review only - http://bmjopen.bmj.com/site/about/guidelines.xhtml

tomato farmers, whose income fell by almost 50% [23]. In another study, structural differences in market infrastructure in different states of India were found to be the reason for different challenges being suffered by farmers across India. For instance, in Haryana state of India, the procurement of crops were at a relatively stable prices as compared to Odisha state of India with improper procurement systems [24].

Our findings are also consistent with experiences from other LMICs. In Bangladesh, about 90% of surveyed households experienced negative income shocks due to their lockdown, and about 88% of the households became food insecure [25]. A study in two East African countries, Kenya and Uganda reported that during the first wave of COVID-19 pandemic the proportion of the food insecure population increased by 38% and 42% respectively, and more than two-third of the population suffered from negative income shocks [26]. A panel data study in Nigeria found an increase of 6-15 percentage points in households experiencing food insecurity when their lockdown was implemented [27].

In this context, our results suggested that the size and impact of negative economic shocks were largest in comparison to other factors affecting food insecurity during the pre-lockdown period. This implies the sensitive nature of food insecurity to economic shocks and intuitively suggests that economic shocks that put stress on livelihoods would have amplified the detrimental effect on food security during the pandemic times as well. To financially cope up with this potential amplified effect of shocks, we found that households adopted strategies such as borrowing either through loan (50%) or from neighbor's, relatives (48%) followed by using personal savings (46%). Other strategies adopted were cutting down on the food consumption (26.3%) and discontinue regular medications or health check-ups (21.5%). All these strategies would probably have long-term repercussions in terms of poor diet quality. Future studies focusing on these aspects should also take these pathways into account.

For peer review only

For peer review only - http://bmjopen.bmj.com/site/about/guidelines.xhtml

Limitations, Strengths and Policy implications

There are some limitations of this study. First, survey 2 was telephonic which could have affected the way the enumerators interpreted responses. Second, all households from Survey 1 could not be followed-up, probably because participants have changed their mobile phone numbers or have migrated to other areas where connectivity may be an issue, but the followed-up household was a representative sub-sample of survey 1.

This study contributes to the documentation and an understanding of the tangible impact of an exogenous economic shock like the COVID-19 lockdown on food insecurity and the role of social safety net programs, such as direct benefit cash transfers in providing a shield to cope with distress situations linked to food and nutrition insecurity in rural India. The findings suggest that effective policy responses are needed to protect food intake in the context of parallel actions that impair food access, to protect food security of the population.

For peer review only

For peer review only - http://bmjopen.bmj.com/site/about/guidelines.xhtml

Contributors

SM and **TT** conceptualized the manuscript and methodology. **SM, SS, SMT, ATJ** and **TT** were responsible for conducting the survey. **SM** conducted the data preparation and analyses. **JRM** contributed to the analyses. **SM** wrote the original draft, reviewed, and edited it based on the comments from all authors. **JRM, SS, SMT, ATJ, PW, AVK** and **TT** reviewed and edited the manuscript. **TT** was responsible for the funding acquisition. All the authors read and approved the final manuscript.

Competing interests

None declared

Funding

This work was supported by the Bill & Melinda Gates Foundation, Seattle, WA [Grant Number: OPP1194964].

Patient consent for publication

Not required

Ethics approval

Ethical approval was obtained for the survey from the Institutional Ethics Committee (IEC reference number: 297/2018) at St. Johns Medical College and Hospital (Bengaluru, India) and permissions from the state government of Bihar were obtained. Verbal informed consent was obtained from the participants prior to data collection for the telephonic survey, while written consent was obtained for the in-person surveys prior to lockdown

Data availability statement

Data are available upon reasonable request.

For peer review only - http://bmjopen.bmj.com/site/about/guidelines.xhtml

References:

1 FAO, IFAD, UNICEF, WFP and WHO. The State of Food Security and Nutrition in the World 2021.Transforming food systems for food security, improved nutrition and affordable healthy diets for all. Rome, FAO: 2021. doi:10.4060/cb4474en

2 Food and Agriculture Organization. Hunger and food insecurity. https://www.fao.org/hunger/en/ (accessed 15 Sep 2021).

3 United Nations. Policy Brief: The Impact of COVID-19 on Food Security and Nutrition. 2020.

4 Lakner C, Yonzan N, Mahler DG, *et al.* Updated estimates of the impact of COVID-19 on global poverty: Looking back at 2020 and the outlook for 2021. Data Blog. 2021.https://blogs.worldbank.org/opendata/updated-estimates-impact-covid-19-global-poverty-looking-back-2020-and-outlook-2021 (accessed 27 Oct 2021).

5 Abhishek, Bhamoriya V, Gupta P, *et al.* India's Food System in the Time of COVID-19. *Econ Polit Wkly* 2020;**55**.

6 Dev SM. Income Support Through Cash Transfers and Employment Guarantee Schemes During the Pandemic Times in India. *Indian J Labour Econ* 2020;**63**:133–8. doi:10.1007/s41027-020-00268-9

7 Vyas M. The jobs bloodbath of April 2020. Cent. Monit. Indian Econ. Pvt. Ltd. 2020.https://www.cmie.com/kommon/bin/sr.php?kall=warticle&dt=2020-05-05 08:22:21&msec=776 (accessed 15 Jul 2021).

8 Tata–Cornell Institute. Pandemic Prices: COVID-19 Price Shocks and Their Implications for Nutrition Security in India. Ithaca, NY: 2020.

9 Ministry of Finance, Government of India. Finance Minister announces Rs 1.70 Lakh

For peer review only - http://bmjopen.bmj.com/site/about/guidelines.xhtml

Crore relief package under Pradhan Mantri Garib Kalyan Yojana for the poor to help them fight the battle against Corona Virus. 2020.https://pib.gov.in/PressReleaseIframePage.aspx?PRID=1608345 (accessed 1 Aug 2021).

10 Bastagli F, Hagen-Zanker J, Harman L, *et al.* Cash transfers: what does the evidence say? A rigorous review of programme impact and of the role of design and implementation features. 2016.

11 Attanasio O, Mesnard A. The Impact of a Conditional Cash Transfer Programme on Consumption in Colombia. *Fisc Stud* 2006;**27**:421–42. doi:10.1111/j.1475-5890.2006.00041.x

12 Maluccio JA. Coping with the "Coffee Crisis" in Central America: The Role of the Nicaraguan Red de Protección Social. FCND discussion papers 188, International Food Policy Research Institute (IFPRI) 2005.

13 FAO. Methods for estimating comparable rates of food insecurity experienced by adults throughout the world. Rome: 2016.

14 Webb P, Coates J, Frongillo EA, *et al.* Measuring Household Food Insecurity: Why It's So Important and Yet So Difficult to Do. *J Nutr* 2006;**136**:1404S-1408S. doi:10.1093/jn/136.5.1404S

15 Coates J, Frongillo EA, Rogers BL, *et al.* Commonalities in the Experience of Household Food Insecurity across Cultures: What Are Measures Missing? *J Nutr* 2006;**136**:1438S-1448S. doi:10.1093/jn/136.5.1438S

16 Sheikomar OB, Dean W, Ghattas H, *et al.* Validity of the Food Insecurity Experience Scale (FIES) for Use in League of Arab States (LAS) and Characteristics of Food

For peer review only

Insecure Individuals by the Human Development Index (HDI). *Curr Dev Nutr* 2021;**5**. doi:10.1093/cdn/nzab017

17 Pereira M, Oliveira AM. Poverty and food insecurity may increase as the threat of COVID-19 spreads. *Public Health Nutr* 2020;**23**:3236–40. doi:10.1017/S1368980020003493

18 Gentilini U. A game changer for social protection? Six reflections on COVID-19 and the future of cash transfers. Let's Talk Development. 2021.https://blogs.worldbank.org/developmenttalk/game-changer-social-protection-six-reflections-covid-19-and-future-cash-transfers (accessed 10 Nov 2021).

19 Filmer D, Friedman J, Kandpal E, *et al.* Cash Transfers, Food Prices, and Nutrition Impacts on Nonbeneficiary Children. Washington, D.C.: 2018. http://documents.worldbank.org/curated/en/989031522077749796/Cash-Transfers-Food-Prices-and-Nutrition-Impacts-on-Nonbeneficiary-Children

20 Attanasio O, Pastorino E. Nonlinear Pricing in Village Economies. *Econometrica* 2020;**88**:207–63. doi:10.3982/ECTA13918

21 Beegle K, Galasso E, Goldberg J. Direct and indirect effects of Malawi's public works program on food security. *J Dev Econ* 2017;**128**:1–23. doi:10.1016/j.jdeveco.2017.04.004

22 Nguyen PH, Kachwaha S, Pant A, *et al.* Impact of COVID-19 on household food insecurity and interlinkages with child feeding practices and coping strategies in Uttar Pradesh, India: a longitudinal community-based study. *BMJ Open* 2021;**11**:e048738. doi:10.1136/bmjopen-2021-048738

23 Ceballos F, Kannan S, Kramer B. Crop prices, farm incomes, and food security during

For peer review only - http://bmjopen.bmj.com/site/about/guidelines.xhtml

the COVID-19 pandemic in India: Phone-based producer survey evidence from Haryana State. *Agric Econ* 2021;**52**:525–42. doi:10.1111/agec.12633

24 Ceballos F, Kannan S, Kramer B. Impacts of a national lockdown on smallholder farmers' income and food security: Empirical evidence from two states in India. *World Dev* 2020;**136**:105069. doi:10.1016/j.worlddev.2020.105069

25 Ahmed F, Islam A, Pakrashi D, *et al.* Determinants and dynamics of food insecurity during COVID-19 in rural Bangladesh. *Food Policy* 2021;**101**. doi:10.1016/j.foodpol.2021.102066

26 Kansiime MK, Tambo JA, Mugambi I, *et al.* COVID-19 implications on household income and food security in Kenya and Uganda: Findings from a rapid assessment. *World Dev* 2021;**137**:105199. doi:10.1016/j.worlddev.2020.105199

27 Amare M, Abay KA, Tiberti L, *et al.* COVID-19 and food security: Panel data evidence from Nigeria. *Food Policy* 2021;**101**. doi:10.1016/j.foodpol.2021.102099

For peer review only - http://bmjopen.bmj.com/site/about/guidelines.xhtml

Figure Legend

Figure1: Framework outlay for the data sources used and methodology adopted. FIES is the Food Insecurity Experience Scale survey which has a set of eight questions with dichotomous responses (Yes or No).

For peer review only

Table 1: Household characteristics in the pre lockdown period (N=1713)

		Median [IQR]/ n(%)[1]
Age (years)		40 [30,58]
Household size		6 [5,8]
Land owned (acres)		0.09 [0.02,0.56]
Household head education	Illiterate	1,052 (61.4%)
	Literate	661 (38.6%)
Household head gender	Male	1,526 (89.1%)
	Female	187 (10.9%)
PDS Beneficiary	Yes	880 (51.4%)
	No	833 (48.6%)
Caste category	Forward	193 (11.3%)
	Other backward classes	980 (57.2%)
	Scheduled castes	540 (31.5%)
Employment category	Casual labour	437 (25.5%)
	Regular Salaried	317 (18.5%)
	Self Employed in Agriculture	767 (44.8%)
	Self Employed in Non-agriculture	192 (11.2%)
Household wealth status[2]	Quintile 1	1027 [902, 1128]/ 348 (20.3%)
	Quintile 2	1429 [1318, 1527]/ 343 (20.0%)
	Quintile 3	1843 [1728, 1956]/ 340 (19.8%)
	Quintile 4	2408 [2257, 2635]/ 341 (19.9%)
	Quintile 5	3923 [3276, 5437]/ 341 (19.9%)

[1]*Numbers are presented as median (inter-quartile range) for continuous measures, and frequency (%) for categorical measures*

For peer review only

[2] *Currency unit for median [inter-quartile range] is given in Indian Rupees (INR); ~75 INR = 1 USD (United States Dollar).*

For peer review only

For peer review only - http://bmjopen.bmj.com/site/about/guidelines.xhtml

Table 2: Prevalence rates of food insecurity

	Survey 1 (N=1713)[1]	Survey 1 subsample (N=859)[2]	Survey 2 (N=859)[3]
Prevalence rate of food insecurity (95% Confidence Interval)	21.5% (19.6 – 23.4)	20.1% (17.4 – 22.8)	47.1% (43.8 – 50.4)

[1]*Survey 1 - Pre lockdown survey*
[2]*Survey 1 subsample-Pre lockdown survey subsample of households followed up in post lockdown survey*
[3]*Survey 2-Post lockdown survey*

For peer review only

Table 3: Factors affecting food security in the pre lockdown period[1] (N=1713)

Food Insecure	Odds Ratio	p-value	95% Confidence Interval
PDS Beneficiary: Yes[2]			
No	1.04	0.75	0.80, 1.35
Land owned (acres)	1.00	0.24	1.00, 1.00
Household size	0.96	0.13	0.90, 1.01
Household head education status: literate[2]			
Illiterate	1.41	0.01	1.08, 1.83
Age (years)	0.99	0.18	0.98, 1.00
Positive or no income shock[2,3]			
Negative Income shock	6.41	<0.001	4.97, 8.27
Employment category: Casual labour[2]			
Regular Salaried	0.65	0.02	0.45, 0.94
Self Employed in Agriculture	0.69	0.02	0.51, 0.95
Self Employed in Non-agriculture	0.77	0.26	0.49, 1.21
MPCE categories[4] : Quintile 1[2]			
Quintile 2	0.89	0.55	0.61, 1.31
Quintile 3	0.85	0.40	0.58, 1.24
Quintile 4	0.63	0.02	0.42, 0.94
Quintile 5	0.66	0.05	0.43, 1.00

[1]*Analysis using logistic regression.*
[2]*Reference category.*
[3]*No or positive economic shocks includes found a job, hike in salary and received food and money as gift whereas negative economic shock includes business closures, mass layoffs, price increase of commodities, job loss, wage cuts, loss of remittances, low rate for produce to be sold at market price, indebtedness, and crop failure.*
[4]*Monthly per capita expenditure quintiles as a proxy for income quintile.*

For peer review only - http://bmjopen.bmj.com/site/about/guidelines.xhtml

Table 4: Factors affecting food security in post lock down period[1] (N=859)

Food Insecure	Odds Ratio	p-value	95% Confidence Interval
Received cash benefit: No[2]			
Yes	0.75	0.047	0.56, 0.99
PDS Beneficiary: Yes[2]			
No	1.05	0.75	0.79, 1.39
Land owned (acres)	0.91	0.16	0.81, 1.04
Household size	1.002	0.94	0.95, 1.05
Household head education status: literate[2]			
Illiterate	1.47	0.01	1.09, 1.99
Age (years)	0.99	0.03	0.98, 1.00
Employment category: Casual labour[2]			
Regular Salaried	0.68	0.08	0.44, 1.05
Self Employed in Agriculture	0.79	0.20	0.55, 1.13
Self Employed in Non-agriculture	0.68	0.12	0.41, 1.11
MPCE categories[3]: Quintile 1[2]			
Quintile 2	0.90	0.63	0.58, 1.40
Quintile 3	0.87	0.53	0.55, 1.36
Quintile 4	1.00	1.00	0.64, 1.56
Quintile 5	0.67	0.09	0.42, 1.07

[1]*Analysis using logistic regression.*
[2]*Reference category.*
[3]*Monthly per capita expenditure quintiles as a proxy for income quintile.*

For peer review only - http://bmjopen.bmj.com/site/about/guidelines.xhtml

For peer review only

Table 5: Cash transfer and Food insecurity for the longitudinal data[1] (N=859)

Food Insecure	Odds Ratio	p value	95% Confidence Interval
Time of assessment: Pre lockdown[2]			
Post Lockdown	4.49	<0.001	3.41, 5.92
Received cash benefits: No[2]			
Yes	1.15	0.43	0.81, 1.61
Time period * Received cash benefits	0.65	0.06	0.42, 1.01
PDS Beneficiary: Yes[2]			
No	1.38	0.41	0.64, 2.98
Land owned (acres)	0.91	0.57	0.67, 1.25
Household size	0.98	0.41	0.93, 1.03
Household head education status: literate[2]			
Illiterate	1.37	0.04	1.02, 1.85
Age (years)	0.99	0.04	0.98, 1.00
Employment category: Casual labour[2]			
Regular Salaried	0.67	0.02	0.48, 0.94
Self Employed in Agriculture	0.70	0.02	0.53, 0.94
Self Employed in Non-agriculture	0.64	0.02	0.43, 0.94
MPCE categories[3]: Quintile 1[2]			
Quintile 2	0.85	0.35	0.60, 1.19
Quintile 3	0.91	0.59	0.64, 1.29
Quintile 4	0.89	0.51	0.62, 1.27
Quintile 5	0.75	0.13	0.51, 1.09
Propensity score	5.93	0.62	0.01, 6794.14

[1]*Analysis using generalized estimation equation model.*
[2]*Reference category.*
[3]*Monthly per capita expenditure quintiles as a proxy for income quintiles.*

For peer review only - http://bmjopen.bmj.com/site/about/guidelines.xhtml

Figure 1: Framework outlay for the data sources used and methodology adopted

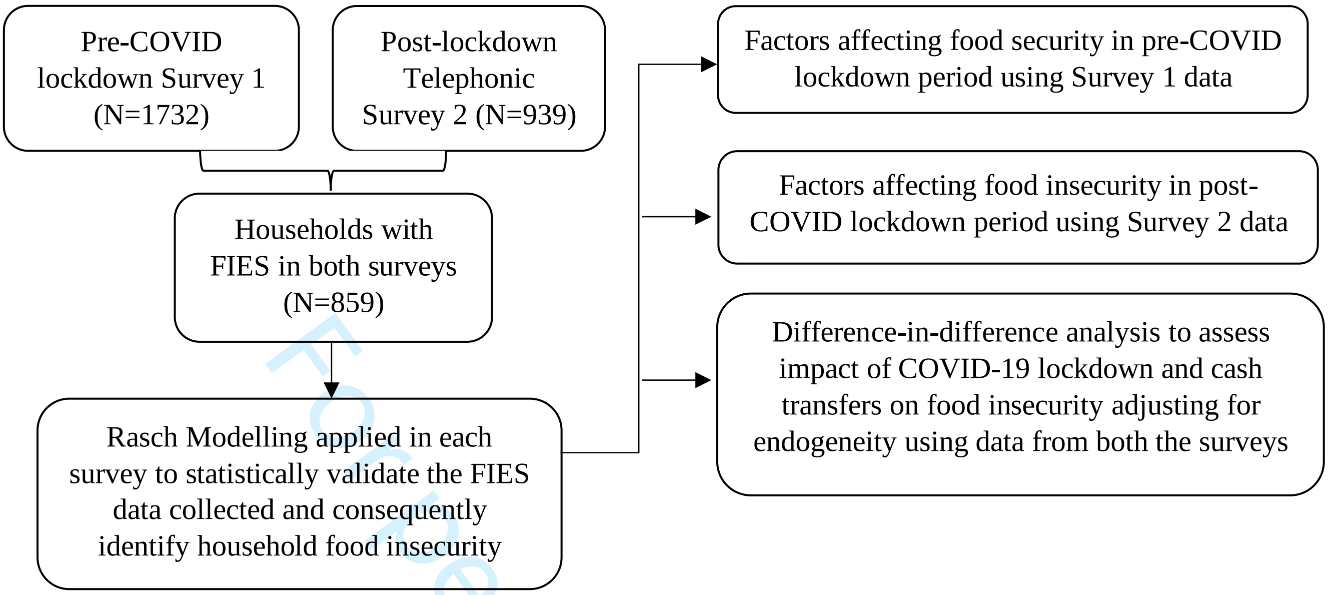

For peer review only

Supplementary Table 1: Household characteristics by cash benefit status in the post lockdown period (N=859)

		Households who did not receive cash benefits (N=497)	Households who received cash benefits (N=362)	p-value
Age(years)		40 [30,58.5]	42 [30,55]	0.79
Household size		7 [5,9]	7 [6,9]	0.078
Land owned (hectares)		0.63 [0.13,2.58]	0.25 [0.08,1.95]	0.016
Household head education	Illiterate	334 (67.2%)	240 (66.3%)	0.78
	Literate	163 (32.8%)	122 (33.7%)	
Household head gender	Male	448 (90.1%)	315 (87.0%)	0.15
	Female	49 (9.9%)	47 (13.0%)	
PDS Beneficiary	Yes	231 (46.5%)	209 (57.7%)	0.001
	No	266 (53.5%)	153 (42.3%)	
Caste category	Forward class	64 (12.9%)	41 (11.3%)	0.79
	Other backward classes	289 (58.1%)	215 (59.4%)	
	Dalit & mahadalits	144 (29.0%)	106 (29.3%)	
Employment category	Casual labourer	117 (23.5%)	91 (25.1%)	0.93
	Regular Salaried	95 (19.1%)	64 (17.7%)	

For peer review only

	Self Employed in Agriculture	229 (46.1%)	166 (45.9%)	
	Self Employed in Non-agriculture	56 (11.3%)	41 (11.3%)	
Household wealth status	Quintile 1	88 (17.7%)	67 (18.5%)	0.66
	Quintile 2	97 (19.5%)	76 (21.0%)	
	Quintile 3	97 (19.5%)	77 (21.3%)	
	Quintile 4	105 (21.1%)	77 (21.3%)	
	Quintile 5	110 (22.1%)	65 (18.0%)	

**Numbers are presented as median (Inter-Quartile range) for continuous measures, and frequency (%) for categorical measures*

For peer review only

STROBE 2007 (v4) Statement—Checklist of items that should be included in reports of *cohort studies*

Section/Topic	Item #	Recommendation	Reported on page #
**Title and abstract**	1	(*a*) Indicate the study's design with a commonly used term in the title or the abstract	1
		(*b*) Provide in the abstract an informative and balanced summary of what was done and what was found	2,3
**Introduction**			
Background/rationale	2	Explain the scientific background and rationale for the investigation being reported	5,6
Objectives	3	State specific objectives, including any prespecified hypotheses	6
**Methods**			
Study design	4	Present key elements of study design early in the paper	6,7
Setting	5	Describe the setting, locations, and relevant dates, including periods of recruitment, exposure, follow-up, and data collection	6,7
Participants	6	(*a*) Give the eligibility criteria, and the sources and methods of selection of participants. Describe methods of follow-up	6,7
		(*b*) For matched studies, give matching criteria and number of exposed and unexposed	N/A
Variables	7	Clearly define all outcomes, exposures, predictors, potential confounders, and effect modifiers. Give diagnostic criteria, if applicable	8-10
Data sources/ measurement	8*	For each variable of interest, give sources of data and details of methods of assessment (measurement). Describe comparability of assessment methods if there is more than one group	6,7
Bias	9	Describe any efforts to address potential sources of bias	9,10
Study size	10	Explain how the study size was arrived at	6,7 (Figure 1)
Quantitative variables	11	Explain how quantitative variables were handled in the analyses. If applicable, describe which groupings were chosen and why	8-10
Statistical methods	12	(*a*) Describe all statistical methods, including those used to control for confounding	8-10 (Figure 1)
		(*b*) Describe any methods used to examine subgroups and interactions	9,10
		(*c*) Explain how missing data were addressed	N/A
		(*d*) If applicable, explain how loss to follow-up was addressed	N/A
		(*e*) Describe any sensitivity analyses	9,10
**Results**			

For peer review only

Participants	13*	(a) Report numbers of individuals at each stage of study—eg numbers potentially eligible, examined for eligibility, confirmed eligible, included in the study, completing follow-up, and analysed	6-7
		(b) Give reasons for non-participation at each stage	7
		(c) Consider use of a flow diagram	Figure 1
Descriptive data	14*	(a) Give characteristics of study participants (eg demographic, clinical, social) and information on exposures and potential confounders	11 (Table 1), 13 (Supplementary Table 1)
		(b) Indicate number of participants with missing data for each variable of interest	N/A
		(c) Summarise follow-up time (eg, average and total amount)	6
Outcome data	15*	Report numbers of outcome events or summary measures over time	Table 2, Table 5
Main results	16	(*a*) Give unadjusted estimates and, if applicable, confounder-adjusted estimates and their precision (eg, 95% confidence interval). Make clear which confounders were adjusted for and why they were included	11-14 (Table 2-5)
		(*b*) Report category boundaries when continuous variables were categorized	Table 1, Supplementary Table 1
		(*c*) If relevant, consider translating estimates of relative risk into absolute risk for a meaningful time period	N/A
Other analyses	17	Report other analyses done—eg analyses of subgroups and interactions, and sensitivity analyses	13,14
**Discussion**			
Key results	18	Summarise key results with reference to study objectives	14
**Limitations**			
Interpretation	20	Give a cautious overall interpretation of results considering objectives, limitations, multiplicity of analyses, results from similar studies, and other relevant evidence	17
Generalisability	21	Discuss the generalisability (external validity) of the study results	15,16
**Other information**			
Funding	22	Give the source of funding and the role of the funders for the present study and, if applicable, for the original study on which the present article is based	18

*Give information separately for cases and controls in case-control studies and, if applicable, for exposed and unexposed groups in cohort and cross-sectional studies.

For peer review only

Note: An Explanation and Elaboration article discusses each checklist item and gives methodological background and published examples of transparent reporting. The STROBE checklist is best used in conjunction with this article (freely available on the Web sites of PLoS Medicine at http://www.plosmedicine.org/, Annals of Internal Medicine at http://www.annals.org/, and Epidemiology at http://www.epidem.com/). Information on the STROBE Initiative is available at www.strobe-statement.org.

For peer review only

The role of cash transfers in mitigating food insecurity in India during the COVID-19 pandemic: a longitudinal study in the Bihar state

Journal:	*BMJ Open*
Manuscript ID	bmjopen-2021-060624.R1
Article Type:	Original research
Date Submitted by the Author:	26-Apr-2022

Complete List of Authors:	Makkar, Sanchit; St John's Research Institute, Division of Nutrition<br>Manivannan, Jawahar ; St John's Research Institute, Division of Epidemiology and Biostatistics<br>Swaminathan, Sumathi; St John's Research Institute, Division of Nutrition<br>Travasso, Sandra ; St John's Research Institute, Division of Epidemiology and Biostatistics<br>John, Anjaly ; St John's Research Institute, Division of Epidemiology and Biostatistics<br>Webb, Patrick ; Tufts University Friedman School of Nutrition Science and Policy<br>Kurpad, Anura; St John's Medical College, Department of Physiology<br>Thomas, Tinku; St John's Medical College, Department of Biostatistics

**Primary Subject Heading**:	Public health
Secondary Subject Heading:	Research methods, Global health
Keywords:	COVID-19, STATISTICS & RESEARCH METHODS, PUBLIC HEALTH

I, the Submitting Author has the right to grant and does grant on behalf of all authors of the Work (as defined in the below author licence), an exclusive licence and/or a non-exclusive licence for contributions from authors who are: i) UK Crown employees; ii) where BMJ has agreed a CC-BY licence shall apply, and/or iii) in accordance with the terms applicable for US Federal Government officers or employees acting as part of their official duties; on a worldwide, perpetual, irrevocable, royalty-free basis to BMJ Publishing Group Ltd ("BMJ") its licensees and where the relevant Journal is co-owned by BMJ to the co-owners of the Journal, to publish the Work in this journal and any other BMJ products and to exploit all rights, as set out in our licence.

The Submitting Author accepts and understands that any supply made under these terms is made by BMJ to the Submitting Author unless you are acting as an employee on behalf of your employer or a postgraduate student of an affiliated institution which is paying any applicable article publishing charge ("APC") for Open Access articles. Where the Submitting Author wishes to make the Work available on an Open Access basis (and intends to pay the relevant APC), the terms of reuse of such Open Access shall be governed by a Creative Commons licence – details of these licences and which Creative Commons licence will apply to this Work are set out in our licence referred to above.

Other than as permitted in any relevant BMJ Author's Self Archiving Policies, I confirm this Work has not been accepted for publication elsewhere, is not being considered for publication elsewhere and does not duplicate material already published. I confirm all authors consent to publication of this Work and authorise the granting of this licence.

For peer review only

The role of cash transfers in mitigating food insecurity in India during the COVID-19 pandemic: a longitudinal study in the Bihar state

Sanchit Makkar[1], Jawahar Ramasamy Manivannan[2], Sumathi Swaminathan[1], Sandra M. Travasso[2], Anjaly Teresa John[2], Patrick Webb[3], Anura V Kurpad[4], Tinku Thomas[5]

[1] Division of Nutrition, St. John's Research Institute, Bengaluru, India

[2] Division of Epidemiology and Biostatistics, St. John's Research Institute, Bengaluru, India

[3] Friedman School of Nutrition Science and Policy, Tufts University, Boston, U.S.A

[4] Department of Physiology, St. John's Medical College, Bengaluru, India

[5] Department of Biostatistics, St. John's Medical College, Bengaluru, India

Corresponding Author:

Dr. Tinku Thomas

Department of Biostatistics,

St. John's Medical College, Bengaluru, India

E-mail: tinku.sarah@sjri.res.in

Word count: 4154

For peer review only - http://bmjopen.bmj.com/site/about/guidelines.xhtml

Abstract

Objective: There are scant empirical data on the impacts of the Coronavirus (COVID-19) pandemic on food security across the globe. India is no exception, with insights into the impacts of lockdown on food insecurity now emerging. We contribute to the empirical evidence on the prevalence of food insecurity in Bihar state before and after lock-down, and whether the government's policy of cash transfer moderated negative effects of food insecurity or not.

Design: This was a longitudinal study.

Settings: The study was conducted in Gaya and Nalanda district of Bihar state in India from December 2019 to September 2020.

Participants: A total of 1797 households were surveyed in Survey 1 and about 52% (n=939) were followed up in survey 2. Valid data for 859 households were considered for the analysis.

Main outcome measures: Using the Food Insecurity Experience Scale (FIES), household conditions were compared pre- and post-lockdown. The effect of cash transfers was examined in a quasi-experimental method using a longitudinal study design. Logistic regression and propensity score adjusted analyses were used to identify factors associated with food insecurity.

Results: Household food insecurity worsened considerably during lockdown, rising from 20% (95% CI:17.4-22.8) to 47% (95% CI: 43.8-50.4) at the sample mean. Households experiencing negative income shocks were more likely to have been food insecure before the lockdown (adjusted Odds Ratio=6.4, 95% CI:4.9-8.3). However, households that received cash transfers had lower odds of being food insecure once the lockdown was lifted (adjusted Odds Ratio=0.75, 95% CI:0.56-0.99).

For peer review only - http://bmjopen.bmj.com/site/about/guidelines.xhtml

Conclusion: These findings provide evidence on how the swift economic response to the pandemic crises using targeted income transfers were relatively successful in mitigating potentially deep impacts of food insecurity.

Keywords:

Cash transfers, COVID-19, Food insecurity, India

For peer review only

For peer review only - http://bmjopen.bmj.com/site/about/guidelines.xhtml

Strengths and limitations of this study

- We studied the effect of COVID -19 pandemic on Household Food Insecurity and role of cash transfers in mitigating this effect if any in the Bihar state of India.
- We used Rasch modelling to statistically validate the food insecurity information collected through longitudinal survey.
- Role of cash transfers were analyzed in a longitudinal study design using quasi-experimental approach after addressing for endogeneity.
- The follow up survey conducted telephonically led to drop out of some households and potentially affected the way enumerators collected information.

For peer review only

1. Introduction

The COVID-19 pandemic is known to have had serious negative effects across the world, with particularly concerning implications for poverty, malnutrition, and food insecurity in low- and middle-income countries (LMICs). However, empirical evidence of such impacts remain scarce [1]. Food insecurity is defined as "when a person lacks regular access to enough safe and nutritious food for normal growth and development and an active and healthy life. This may be due to unavailability of food and/or lack of resources to obtain food. Food insecurity can be experienced at different levels of severity" [2]. It is estimated that in South and Southeast Asia, around 33 million people may have been pushed into a state of acute food insecurity since February 2020 [3], and that between 88 and 115 million people globally were pushed into extreme poverty during 2020 [4].

While governments scrambled to curb the spread of the disease, one of the most common policy measures was to prevent the movement and congregation of individuals by imposing lockdowns that were intended to isolate vulnerable people and prevent transmission. An unfortunate (but foreseen) side-effect of lockdown was increased poverty and constrained access to food due to an inability to leave the home, and because of loss of jobs and income. In India, a rigorous lockdown imposed on 23rd March 2020 brought most economic activities to a halt. The abrupt lockdown caused disruptions to food supplies, restricted labor availability and severely cut incomes, resulting in an economic downturn that affected most of India's 1.3 billion population [5]. A survey by the Centre for Monitoring Indian Economy (CMIE) showed a steep rise in unemployment rate across India from 8.4% in March 2020 to 23.5% in April 2020, with 75% (91 million) attributed to job loss among small traders and casual labor [6,7]. During the period of lockdown, the prices for non-cereal nutrient-dense foods (pulses, vegetables and eggs) rose faster and higher than the price of cereal foods like wheat and rice [8]. In addition, the government's long-standing supplementary feeding programs, specifically

For peer review only - http://bmjopen.bmj.com/site/about/guidelines.xhtml

the Integrated Child Development Services and the Mid-day Meal Program, were suspended, which negatively impacted food consumption of entitled children and women. In an attempt to mitigate the expected adverse effects of the lockdown, the government implemented an urgent relief package valued at INR 1.7 lakh crore (~US$22.6 billion), including both cash and food support [9]. This paper focuses on the cash transfers and its effect on food insecurity.

Cash transfers can play a significant role in diversifying the diet and improving household food consumption [10,11], because it can smooth the variability of food consumption by stabilizing income, thereby protecting food intake at household level [12]. However, the impact of cash transfer initiative specific to COVID-19 has not been studied so far. Intuitively, financial assistance provided in response to COVID-19 through cash transfer instruments would have protected household purchasing power in the face of price hikes. Therefore, cash transfers would have buffered against rising food insecurity at the household level. In this context, our specific objective was to document the state of food insecurity in two districts of Bihar and the role (if any) of cash transfers in mitigating negative impacts on food insecurity at the household level.

2. Methodology

Sources of Data

We conducted a longitudinal study in Gaya and Nalanda districts in Bihar. A door-to-door survey was conducted from July 2019 through September 2019, and a follow-up (denoted as Survey 1) survey was undertaken from December 2019 through February 2020 (n=1732 households). Of 1797 households contacted, valid data were available for 1732. The original intent was to examine linkages between agriculture and nutrition in rural areas, with a focus on five nutrient-dense foods: namely, pulses (red lentils), milk, green leafy vegetables, eggs and poultry. A total of 142 villages in Gaya district and 134 in Nalanda district were sampled

For peer review only - http://bmjopen.bmj.com/site/about/guidelines.xhtml

through multistage cluster sampling, and households were chosen by the random walk method (10 households per village). To account for heterogeneity in distances to district market centers, the villages were sampled from 0-5 km, 6-15 km, 16-30 km, and >30 km distance bands from the district headquarters. Within each village, households involved in the production of the nutrient-dense foods, landholding households (regardless of their output), and landless households were included in the sampling frame. Data on household consumption of food in the previous month was collected from all households, and information related to food production was collected from those households involved in the production of nutrient-dense foods.

When lockdown was eased, a telephonic survey (Survey 2) was conducted in the same households during August and September 2020. Using the contact numbers (1797 households) provided during Survey 1, all respondents were contacted telephonically. After multiple attempts, response rate was 52.3% (n=939) with >50% response rate in each district. The aim of the telephonic survey was to identify the effects of the pandemic on food production and consumption in the context of restrictions. All interviews were audio recorded, as well as documented in interviewer notes. The head of the household or his/her spouse were the respondents and the interviews were in Hindi.

The survey instrument included questions on the effects of COVID-19 on participant income/livelihoods, effects on food production and sale (among food producing households), support received in cash and in-kind from the government, NGOs and others during the period, and effects on food purchase and consumption (among all respondents). The widely validated Food Insecurity Experience Scale (FIES) that determines food access at different levels of severity was also administered. Complete FIES data were available from 1713 households in Survey 1 and 859 in Survey 2 were considered for the analysis. **Figure 1** provides the framework, laying out the data sources used, and methodology adopted for the study.

For peer review only - http://bmjopen.bmj.com/site/about/guidelines.xhtml

The Food Insecurity Experience Scale (FIES)

The household version of the Food Insecurity Experience Scale (FIES) survey was administered to all sampled households in survey 1 and 2. The FIES has a set of eight questions with dichotomous responses (Yes or No). Specifically, to determine access to food over the prior month, questions whether the household; a) worried about not having enough food, b) was unable to eat healthy and nutritious food, c) ate only few kinds of foods, d) skipped a meal, e) ate less, f) ran out of food, g) was hungry but did not eat and h) went without eating for a whole day [13] were asked. A Rasch model was used to assess the appropriateness of the FIES data collected in the context of the current study, as recommended by FAO [13]. The model was used to statistically validate the quality of the data based on assumptions such as (1) more severe items are less frequently affirmed and (2) household that affirm a particular item on the scale are likely to affirm lesser severe items on the scale. Although these are not necessary conditions, testing of these served as a validation for the item scoring. The Rasch model is a single parameter logistic item response theory model and was used to validate FIES based on these assumptions, building on the precursor Household Food Insecurity and Access Scale (HFIAS) [14]. The domains identified as significant components of food insecurity are comparable across ethnographic groups [15] and therefore establishes the appropriateness of the tool for the setting.

The four different types of results generated from the Rasch model, which are used to check the assumptions made, are fit statistics which are infit and outfit, residual correlation matrix and Rasch reliability criteria. Infit statistic measures the discriminatory power of an item i.e., items that did not perform well within the given data. The outfit statistic is like infit, but it is sensitive to responses which are extremely unexpected or outliers in the response pattern. To validate the data, a set of criteria for these measures were followed [13,16]. The adequate fit value for both fit statistics had to lie between 0.7 to 1.3. If an outfit value was >2,

For peer review only - http://bmjopen.bmj.com/site/about/guidelines.xhtml

it was considered high. However, if infit statistics were well within range then high outfit values were not considered for eliminating the items. The residual correlation matrix for each pair of items indicates pairs that are redundant and an absolute value > 0.4 was considered high indicating multidimensionality between pair of items. The last assumption (i.e., Rasch Reliability) accounts for the total variance explained by the model. For 8 items FIES scale, a value greater than 0.7 is considered reliable and adequate.

Following statistical validation, the households having a raw score ≤ 3 [16] were identified to be 'food secure'. This category would also include mild food insecure households. The households were categorized to be food insecure for a raw score >3. This binary classification of food insecurity status was used as the outcome variable in the entire analysis.

Statistical Methods

Household factors associated with food insecurity were identified through logistic regressions using the Survey 1 data on 1713 households. The effect of cash transfers on food insecurity was examined in Survey 2 (n=859) data using logistic regression after adjusting for all household factors identified to be associated with food insecurity in Survey 1 analysis. However, cash transfers were based on an eligibility criterion, which implies a non-randomization in sample selection. Therefore, it may cause endogeneity that could arise due to household characteristics being conducive to receiving cash benefit and hence a potential selection bias leading to biased estimate and standard errors with narrower confidence interval.

Further, as a sensitivity analysis to account for potential endogeneity, we computed the propensity score for receipt of cash benefit considering household characteristics that were either associated with cash benefit or with household food insecurity. The variables considered for the computation of the propensity score were: having a ration card or beneficiary of Public Distribution System (PDS) that provides food and non-food items through fair price shops at a

For peer review only - http://bmjopen.bmj.com/site/about/guidelines.xhtml

subsidized rates (yes vs no), household size, land owned (acres), education status (literate vs illiterate) and age of the household head, type of economic activity household is involved (casual labor, regular salaried, self-employed in agriculture and non-agriculture) and wealth quintiles from monthly per capita expenditure.

The difference in change in the odds of being food insecure between Survey 1 to Survey 2 (for households that received and did not receive cash benefits) was examined as an interaction effect between time (pre-lockdown survey and post-lockdown survey) and receipt of cash transfer, in a random effects model with logit link function, using the propensity score as a covariate. The following generalized estimating linear model was used,

$$\text{Logit}(p_{ij}) = \beta_0 + \beta_1 C_{ij} + \beta_2 T_{ij} + \beta_3 (C_{ij} * T_{ij}) + B_4 P_{ij} + \beta_5 X_{ij} + \epsilon_{ij}$$

Where p_{ij} is the probability of household food insecurity for household i in time j where j is 0 (Survey 1) and 1 (Survey 2); C_{ij} is an indicator variable for the household i having received cash benefit in time j; T_{ij} is the time dummy being equal to 0 for pre lock down period and 1 for post lock down period for household i; β_1 is the population average difference in log odds of food insecurity by cash benefit and β_2 is the population average difference in log odds of food insecurity between the two rounds of survey; $C_{ij}*T_{ij}$ is the interaction between the time and receipt of cash transfer. Statistical significance of interaction effect was considered at $p<0.1$. The coefficient β_3 gives the estimated effect of cash transfer on food insecurity over time; P_{ij} is the propensity score for each household; X_{ij} is a matrix containing household characteristics in time j and ϵ_{ij} is the error term.

All results are expressed as odds ratio (95% confidence interval). All analysis were performed using STATA version 16.1.

Patient and Public Involvement

No Patient involved

For peer review only - http://bmjopen.bmj.com/site/about/guidelines.xhtml

3. Results

Socioeconomic characteristics of population before lockdown

Data from 1713 households were included for this analysis (**Table 1**). The median household size was 6 [IQR 5,8] and 11% were women-headed households. Overall, only 39% of household heads were literate, of whom 80% were male. The median area of land owned by households was 0.09 acres. About 51% of the households reported having a ration card, implying that they were beneficiaries of safety net program, the Public Distribution System (PDS). About 25% of all households reported that they were engaged in casual labor, while about 18% had regular salaries, and 56% were self-employed in agriculture and non-agriculture economic activity.

Statistical validation of food insecurity using Rasch modelling

The infit statistics were examined separately in the subgroup of households who participated in both surveys, and therefore made up the longitudinal panel (N=859). The infit statistics were adequately within the range i.e., 0.7 to 1.3, suggesting that the assumption of equal discrimination is met, and all the items are related to the latent trait. However, items such as 'worried' and 'healthy' had the outfit statistic > 2 in both surveys. Since the infit statistics were within range, these items were not dropped based on the outfit measure. Hence, all the 8 items were retained to calculate the prevalence of food insecurity. The overall fit of the model i.e., Rasch reliability criteria (0.74 and 0.71 for Survey 1 and Survey 2 respectively) was greater than 0.7, suggesting a reasonably good fit. The residual correlations between pairs of items were all <0.4 implying no conditional dependence between the pairs of items. In addition, we checked these assumptions for the entire Survey 1 sample (N=1713) and similar results were obtained. The raw scores from all 8 items were used to classify households as food insecure (>3) or not in the two surveys.

For peer review only - http://bmjopen.bmj.com/site/about/guidelines.xhtml

Prevalence of food insecurity pre- and post-lockdown

The prevalence of food insecurity before lockdown was similar in the subsample that participated in Survey 2 (20.1%) as in the entire sample of Survey 1 (21.5%) (**Table 2**). This implies that the Survey 2 sample (n=859) was representative of the population covered in Survey 1 (**Table 2**). The sub-sample that participated in Survey 2 and did not participate were comparable in all other household characteristics as well (**Supplementary Table 1**), except literacy of head of household. Households with illiterate heads were less likely to participate in Survey 2. The prevalence of food insecurity in the sub-sample from Survey 1 increased significantly from 20.1% pre-lockdown to 47.1% post-lockdown ($P < 0.001$), underlining the immediate impact of COVID-19 on the food basket of households.

Factors affecting food insecurity before lockdown

In the pre-lockdown survey of 1713 households (**Table 3**), the literacy of the household head was significantly associated with food insecurity (adjusted OR: 1.41, 95%CI: 1.08, 1.83). Households that had experienced a recent negative economic shock, such as job loss or fall in income, were 6.4 times more likely to suffer food insecurity compared to households that reported either no negative or positive economic shocks, including the finding of a new job (adjusted OR: 6.41, 95%CI: 4.97, 8.27). PDS beneficiary status was not associated with food insecurity status. People engaged in a regular salaried profession or self-employment in agriculture had lower odds of experiencing food insecurity (adjusted OR: 0.65, 95%CI: 0.45, 0.94 and adjusted OR: 0.69, 95%CI: 0.5, 0.95, respectively) compared to casual labourers. Belonging to a higher income class i.e., top 40% (quintile 4 and quintile 5), had lower odds of being food insecure as compared to bottom 20% income class (adjusted OR: 0.63, 95%CI: 0.42, 0.94 and adjusted OR: 0.66, 95%CI: 0.43, 1.00, respectively).

Factors affecting food insecurity over time, including cash transfers

For peer review only - http://bmjopen.bmj.com/site/about/guidelines.xhtml

In the 859 households surveyed in the post-lockdown period, about 42% households had received a cash transfer from the government. The total amount received during the 3 lockdown months (April to June 2020) ranged from INR 200 to INR 7500, with households reporting the receipt of a median INR 1500 [IQR: INR 1000, INR 1600]. In addition, 55% of the households also received food as well as other in-kind benefits from various Non-Governmental Organizations (NGOs), and 28% received both cash and in-kind benefits.

Households that received cash transfers had a lower proportion of food insecurity (43.6%) compared to those who did not (49.7%). Among factors that influenced food security in the post lockdown period (**Table 4**), households that received the transfers were 25% less likely to be food insecure compared to those that did not (adjusted OR: 0.75, 95%CI: 0.56, 0.99). Other factors such as type of employment, wealth quintiles were associated with food insecurity in the same manner as the pre-lockdown period. The lockdown related factors reported by households that could have contributed to food insecurity were restrictions in agricultural activities (42.3%), increased purchase prices (22%-53% depending on the type of food) and negative effect on livelihood (79.3%).

To arrive at better estimates of the effects of cash transfer on food insecurity, longitudinal data (pre and post lockdown surveys) of 859 household were considered. The households that received cash transfer were compared with those who did not. The proportion of PDS beneficiary households were 58% in the group that received the cash transfer (**Supplementary Table 2**) compared to 47% in the group that did not receive this. All other characteristics were comparable between the groups. Therefore, cash benefits could be conditional on eligibility criteria which we could not assess directly. Hence, we also corrected for endogeneity arising due to selection bias by propensity scores adjusted analysis (**Table 5**). The interaction effect of time (pre- vs post-lockdown) and receipt of cash transfer (received cash benefits or not received) was examined to quantify the differential effect of cash benefit

For peer review only - http://bmjopen.bmj.com/site/about/guidelines.xhtml

on household food insecurity change due to the lockdown. Overall, there was a significant increase in household food insecurity in the post-lockdown survey compared to pre-lockdown (adjusted OR: 4.5, 95%CI: 3.4, 5.93). The interaction of time and cash benefit receipt was significant (adjusted OR: 0.65, 95%CI: 0.42, 1.01, p=0.06) such the odds of becoming food insecure in the lockdown period compared to pre-COVID, by the group that received cash benefits in the post lockdown period was only 0.65 times compared to the households that did not receive cash benefits. The adjusted OR was 0.65, 95% CI: 0.44,0.97, p=0.03 after accounting for clustering effects of village. This underscored a beneficial effect of cash transfer on food insecurity even after adjusting for differences in familial characteristics between the households that received and did not receive cash benefits.

4. Discussion

This study contributes evidence on the impact of the COVID-19 pandemic related lockdown on food security in Bihar state, in two ways. First, it examines the status of food insecurity in the pre- and post- lockdown periods. Second, it examines the impact of cash transfers during this period on post- lockdown food insecurity. Our longitudinal survey findings suggest that there was a significant increase in the prevalence of food insecurity immediately post-lockdown, such that this increased steeply, from 20% (pre- lockdown) to 47% (post-lockdown). We also found that the social safety net programs such as cash transfers introduced under the Pradhan Mantri Garib Kalyan Yojana (PM-GKY), had the potential to address this stressful situation, such that the odds of being food insecure in a given household sample were much lower in comparison to households that did not receive the cash transfer. The transfers may have acted as an added income for the households, such that their purchasing power to spend on essential items did not reduce. The paper also examines how the cash transfer mechanism was beneficial to some degree to tackle this crisis.

For peer review only - http://bmjopen.bmj.com/site/about/guidelines.xhtml

Intervention strategies focused on reducing poverty and food insecurity in the context of COVID-19 have been widely implemented across LMICs, but not really assessed in terms of their impacts [17,18]. According to World Bank study, a total of 277 cash transfers programs in 131 countries were introduced to handle COVID-19 crisis by June, 2020 [19]. For example, India announced a relief package in March 2020 as an immediate response to the economic crisis associated with its policy decision to lockdown. Although these existing schemes were important, they did not ensure protection of the various dimensions that affect livelihoods and consequently, food affordability.

The supplementary nutrition such as Integrated Child Development Services, Mid-day meal programs etc. were disrupted during the lockdown period and these programs go a long way in providing quality nutrition to the beneficiary population. This study shows that a cash transfer of approximately INR 500/month was helpful in mitigating the negative effect of the lockdown on food insecurity and was helpful towards meeting the energy requirement of the recipient household. However, the sufficiency of this amount to meet the requirements for dietary diversity of the beneficiary households is still questionable. Some evidence suggests that cash transfers increase local food prices, steepened the price slope and potentially worsened food security for those who were ineligible for transfers [20–22] but this was not investigated in the current study. Our research was limited to understanding the immediate impact of cash transfers on household food insecurity. In the long run, even after the lifting of the COVID-19 lockdown, the bigger problem is the need to holistically meet the nutritional requirements of the population, rather than simply their energy requirements or averting hunger. This is because the revival of the economic status of the population is likely to take longer, especially with COVID-19 related restrictions still persisting, and with repeated waves of infection.

For peer review only - http://bmjopen.bmj.com/site/about/guidelines.xhtml

The effect of the pandemic on food insecurity found in this study is externally valid in India, as a study in Uttar Pradesh state of India showed findings consistent with the present Bihar study, where household food insecurity increased from 21% in December 2019 to 80% August 2020 and children in these households were less likely to consume a diverse diet [23]. As far as food security among the Indian farmers is concerned, a study on tomato and wheat producers showed that the latter were relatively less affected in terms of decline in income due to fixed market prices and hence, were less affected by food insecurity in comparison to the tomato farmers, whose income fell by almost 50% [24]. In another study, structural differences in market infrastructure in different states of India were found to be the reason for different challenges being suffered by farmers across India. For instance, in Haryana state of India, the procurement of crops were at a relatively stable prices as compared to Odisha state of India with improper procurement systems [25].

Our findings are also consistent with experiences from other LMICs. In Bangladesh, about 90% of surveyed households experienced negative income shocks due to their lockdown, and about 88% of the households became food insecure [26]. A study in two East African countries, Kenya and Uganda reported that during the first wave of COVID-19 pandemic the proportion of the food insecure population increased by 38% and 42% respectively, and more than two-third of the population suffered from negative income shocks [27]. A panel data study in Nigeria found an increase of 6-15 percentage points in households experiencing food insecurity when their lockdown was implemented [28]. In Nepal, food insecurity was a serious concern among the low-income and disadvantaged families during the COVID-19, and it severely affected their health and wellbeing [29].

In this context, our results suggested that the size and impact of negative economic shocks were largest in comparison to other factors affecting food insecurity during the pre-lockdown period. This implies the sensitive nature of food insecurity to economic shocks and

For peer review only - http://bmjopen.bmj.com/site/about/guidelines.xhtml

intuitively suggests that economic shocks that put stress on livelihoods would have amplified the detrimental effect on food security during the pandemic times as well. To financially cope up with this potential amplified effect of shocks, we found that households adopted strategies such as borrowing either through loan (50%) or from neighbor's, relatives (48%) followed by using personal savings (46%). Other strategies adopted were cutting down on the food consumption (26.3%) and discontinue regular medications or health check-ups (21.5%). All these strategies would probably have long-term repercussions in terms of poor diet quality. Future studies focusing on these aspects should also take these pathways into account.

Limitations, Strengths and Policy implications

There are some limitations of this study. First, survey 2 was telephonic which could have affected the way the enumerators interpreted responses. Second, all households from Survey 1 could not be followed-up, probably because participants have changed their mobile phone numbers or have migrated to other areas where connectivity may be an issue, but the followed-up household was a representative sub-sample of survey 1.

This study contributes to the documentation and an understanding of the tangible impact of an exogenous economic shock like the COVID-19 lockdown on food insecurity and the role of social safety net programs, such as direct benefit cash transfers in providing a shield to cope with distress situations linked to food and nutrition insecurity in rural India. The findings suggest that effective policy responses are needed to protect food intake in the context of parallel actions that impair food access, to protect food security of the population.

For peer review only - http://bmjopen.bmj.com/site/about/guidelines.xhtml

Contributors

SM and **TT** conceptualized the manuscript and methodology. **SM, SS, SMT, ATJ** and **TT** were responsible for conducting the survey. **SM** conducted the data preparation and analyses. **JRM** contributed to the analyses. **SM** wrote the original draft, reviewed, and edited it based on the comments from all authors. **JRM, SS, SMT, ATJ, PW, AVK** and **TT** reviewed and edited the manuscript. **TT** was responsible for the funding acquisition. All the authors read and approved the final manuscript.

Competing interests

None declared

Funding

This work was supported by the Bill & Melinda Gates Foundation, Seattle, WA [Grant Number: OPP1194964].

Patient consent for publication

Not required

Ethics approval

Ethical approval was obtained for the survey from the Institutional Ethics Committee (IEC reference number: 297/2018) at St. Johns Medical College and Hospital (Bengaluru, India) and permissions from the state government of Bihar were obtained. Verbal informed consent was obtained from the participants prior to data collection for the telephonic survey, while written consent was obtained for the in-person surveys prior to lockdown

Data availability statement

Data are available upon reasonable request.

For peer review only

For peer review only - http://bmjopen.bmj.com/site/about/guidelines.xhtml

References:

1 FAO, IFAD, UNICEF, WFP and WHO. The State of Food Security and Nutrition in the World 2021.Transforming food systems for food security, improved nutrition and affordable healthy diets for all. Rome, FAO: 2021. doi:10.4060/cb4474en

2 Food and Agriculture Organization. Hunger and food insecurity. https://www.fao.org/hunger/en/ (accessed 15 Sep 2021).

3 United Nations. Policy Brief: The Impact of COVID-19 on Food Security and Nutrition. 2020.

4 Lakner C, Yonzan N, Mahler DG, *et al.* Updated estimates of the impact of COVID-19 on global poverty: Looking back at 2020 and the outlook for 2021. Data Blog. 2021. https://blogs.worldbank.org/opendata/updated-estimates-impact-covid-19-global-poverty-looking-back-2020-and-outlook-2021 (accessed 27 Oct 2021).

5 Abhishek, Bhamoriya V, Gupta P, *et al.* India's Food System in the Time of COVID-19. *Econ Polit Wkly* 2020;**55**.

6 Dev SM. Income Support Through Cash Transfers and Employment Guarantee Schemes During the Pandemic Times in India. *Indian J Labour Econ* 2020;**63**:133–8. doi:10.1007/s41027-020-00268-9

7 Vyas M. The jobs bloodbath of April 2020. Cent. Monit. Indian Econ. Pvt. Ltd. 2020. https://www.cmie.com/kommon/bin/sr.php?kall=warticle&dt=2020-05-05 08:22:21&msec=776 (accessed 15 Jul 2021).

8 Tata–Cornell Institute. Pandemic Prices: COVID-19 Price Shocks and Their Implications for Nutrition Security in India. Ithaca, NY: 2020.

9 Ministry of Finance, Government of India. Finance Minister announces Rs 1.70 Lakh

For peer review only - http://bmjopen.bmj.com/site/about/guidelines.xhtml

Crore relief package under Pradhan Mantri Garib Kalyan Yojana for the poor to help them fight the battle against Corona Virus. 2020.

10 Bastagli F, Hagen-Zanker J, Harman L, *et al.* Cash transfers: what does the evidence say? A rigorous review of programme impact and of the role of design and implementation features. 2016.

11 Attanasio O, Mesnard A. The Impact of a Conditional Cash Transfer Programme on Consumption in Colombia. *Fisc Stud* 2006;**27**:421–42. doi:10.1111/j.1475-5890.2006.00041.x

12 Maluccio JA. Coping with the "Coffee Crisis" in Central America: The Role of the Nicaraguan Red de Protección Social. FCND discussion papers 188, International Food Policy Research Institute (IFPRI) 2005.

13 FAO. Methods for estimating comparable rates of food insecurity experienced by adults throughout the world. Rome: 2016.

14 Webb P, Coates J, Frongillo EA, *et al.* Measuring Household Food Insecurity: Why It's So Important and Yet So Difficult to Do. *J Nutr* 2006;**136**:1404S-1408S. doi:10.1093/jn/136.5.1404S

15 Coates J, Frongillo EA, Rogers BL, *et al.* Commonalities in the Experience of Household Food Insecurity across Cultures: What Are Measures Missing? *J Nutr* 2006;**136**:1438S-1448S. doi:10.1093/jn/136.5.1438S

16 Sheikomar OB, Dean W, Ghattas H, *et al.* Validity of the Food Insecurity Experience Scale (FIES) for Use in League of Arab States (LAS) and Characteristics of Food Insecure Individuals by the Human Development Index (HDI). *Curr Dev Nutr* 2021;**5**. doi:10.1093/cdn/nzab017

For peer review only

17 Pereira M, Oliveira AM. Poverty and food insecurity may increase as the threat of COVID-19 spreads. *Public Health Nutr* 2020;**23**:3236–40. doi:10.1017/S1368980020003493

18 Gentilini U. A game changer for social protection? Six reflections on COVID-19 and the future of cash transfers. Let's Talk Development. 2021. https://blogs.worldbank.org/developmenttalk/game-changer-social-protection-six-reflections-covid-19-and-future-cash-transfers (accessed 10 Nov 2021).

19 Gentilini U, Almenfi M, Orton I, *et al.* Social Protection and Jobs Responses to COVID-19 : A Real-Time Review of Country Measures. Washington, DC: 2020. https://openknowledge.worldbank.org/handle/10986/33635

20 Filmer D, Friedman J, Kandpal E, *et al.* Cash Transfers, Food Prices, and Nutrition Impacts on Nonbeneficiary Children. Washington, D.C.: 2018. http://documents.worldbank.org/curated/en/989031522077749796/Cash-Transfers-Food-Prices-and-Nutrition-Impacts-on-Nonbeneficiary-Children

21 Attanasio O, Pastorino E. Nonlinear Pricing in Village Economies. *Econometrica* 2020;**88**:207–63. doi:10.3982/ECTA13918

22 Beegle K, Galasso E, Goldberg J. Direct and indirect effects of Malawi's public works program on food security. *J Dev Econ* 2017;**128**:1–23. doi:10.1016/j.jdeveco.2017.04.004

23 Nguyen PH, Kachwaha S, Pant A, *et al.* Impact of COVID-19 on household food insecurity and interlinkages with child feeding practices and coping strategies in Uttar Pradesh, India: a longitudinal community-based study. *BMJ Open* 2021;**11**:e048738. doi:10.1136/bmjopen-2021-048738

For peer review only - http://bmjopen.bmj.com/site/about/guidelines.xhtml

24 Ceballos F, Kannan S, Kramer B. Crop prices, farm incomes, and food security during the COVID-19 pandemic in India: Phone-based producer survey evidence from Haryana State. *Agric Econ* 2021;**52**:525–42. doi:10.1111/agec.12633

25 Ceballos F, Kannan S, Kramer B. Impacts of a national lockdown on smallholder farmers' income and food security: Empirical evidence from two states in India. *World Dev* 2020;**136**:105069. doi:10.1016/j.worlddev.2020.105069

26 Ahmed F, Islam A, Pakrashi D, *et al.* Determinants and dynamics of food insecurity during COVID-19 in rural Bangladesh. *Food Policy* 2021;**101**. doi:10.1016/j.foodpol.2021.102066

27 Kansiime MK, Tambo JA, Mugambi I, *et al.* COVID-19 implications on household income and food security in Kenya and Uganda: Findings from a rapid assessment. *World Dev* 2021;**137**:105199. doi:10.1016/j.worlddev.2020.105199

28 Amare M, Abay KA, Tiberti L, *et al.* COVID-19 and food security: Panel data evidence from Nigeria. *Food Policy* 2021;**101**. doi:10.1016/j.foodpol.2021.102099

29 Singh DR, Sunuwar DR, Shah SK, *et al.* Food insecurity during COVID-19 pandemic: A genuine concern for people from disadvantaged community and low-income families in Province 2 of Nepal. *PLoS One* 2021;**16**:1–20. doi:10.1371/journal.pone.0254954

For peer review only

For peer review only - http://bmjopen.bmj.com/site/about/guidelines.xhtml

Figure Legend

Figure1: Framework outlay for the data sources used and methodology adopted. FIES is the Food Insecurity Experience Scale survey which has a set of eight questions with dichotomous responses (Yes or No).

For peer review only

Table 1: Household characteristics in the pre lockdown period (N=1713)

		Median [IQR]/ n(%)[1]
Age (years)		40 [30,58]
Household size		6 [5,8]
Land owned (acres)		0.09 [0.02,0.56]
Household head education	Illiterate	1,052 (61.4%)
	Literate	661 (38.6%)
Household head gender	Male	1,526 (89.1%)
	Female	187 (10.9%)
PDS Beneficiary	Yes	880 (51.4%)
	No	833 (48.6%)
Caste category	Forward	193 (11.3%)
	Other backward classes	980 (57.2%)
	Scheduled castes	540 (31.5%)
Employment category	Casual labour	437 (25.5%)
	Regular Salaried	317 (18.5%)
	Self Employed in Agriculture	767 (44.8%)
	Self Employed in Non-agriculture	192 (11.2%)
Household wealth status[2]	Quintile 1	1027 [902, 1128]/ 348 (20.3%)
	Quintile 2	1429 [1318, 1527]/ 343 (20.0%)
	Quintile 3	1843 [1728, 1956]/ 340 (19.8%)
	Quintile 4	2408 [2257, 2635]/ 341 (19.9%)
	Quintile 5	3923 [3276, 5437]/ 341 (19.9%)

[1]Numbers are presented as median (inter-quartile range) for continuous measures, and frequency (%) for categorical measures

For peer review only

For peer review only - http://bmjopen.bmj.com/site/about/guidelines.xhtml

[2] *Currency unit for median [inter-quartile range] is given in Indian Rupees (INR); ~75 INR = 1 USD (United States Dollar).*

For peer review only

For peer review only - http://bmjopen.bmj.com/site/about/guidelines.xhtml

Table 2: Prevalence rates of food insecurity

	Survey 1 (N=1713)[1]	Survey 1 subsample (N=859)[2]	Survey 2 (N=859)[3]
Prevalence rate of food insecurity (95% Confidence Interval)	21.5% (19.6 – 23.4)	20.1% (17.4 – 22.8)	47.1% (43.8 – 50.4)

[1]*Survey 1 - Pre lockdown survey*
[2]*Survey 1 subsample-Pre lockdown survey subsample of households followed up in post lockdown survey*
[3]*Survey 2-Post lockdown survey*

For peer review only

Table 3: Factors affecting food security in the pre lockdown period[1] (N=1713)

Food Insecure	Odds Ratio	p-value	95% Confidence Interval
PDS Beneficiary: Yes[2]			
No	1.04	0.75	0.80, 1.35
Land owned (acres)	1.00	0.24	1.00, 1.00
Household size	0.96	0.13	0.90, 1.01
Household head education status: literate[2]			
Illiterate	1.41	0.01	1.08, 1.83
Age (years)	0.99	0.18	0.98, 1.00
Positive or no income shock[2,3]			
Negative Income shock	6.41	<0.001	4.97, 8.27
Employment category: Casual labour[2]			
Regular Salaried	0.65	0.02	0.45, 0.94
Self Employed in Agriculture	0.69	0.02	0.51, 0.95
Self Employed in Non-agriculture	0.77	0.26	0.49, 1.21
MPCE categories[4] : Quintile 1[2]			
Quintile 2	0.89	0.55	0.61, 1.31
Quintile 3	0.85	0.40	0.58, 1.24
Quintile 4	0.63	0.02	0.42, 0.94
Quintile 5	0.66	0.05	0.43, 1.00

[1]*Analysis using logistic regression.*
[2]*Reference category.*
[3]*No or positive economic shocks includes found a job, hike in salary and received food and money as gift whereas negative economic shock includes business closures, mass layoffs, price increase of commodities, job loss, wage cuts, loss of remittances, low rate for produce to be sold at market price, indebtedness, and crop failure.*
[4]*Monthly per capita expenditure quintiles as a proxy for income quintile.*

For peer review only

For peer review only - http://bmjopen.bmj.com/site/about/guidelines.xhtml

Table 4: Factors affecting food security in post lock down period[1] (N=859)

Food Insecure	Odds Ratio	p-value	95% Confidence Interval
Received cash benefit: No[2]			
Yes	0.75	0.047	0.56, 0.99
PDS Beneficiary: Yes[2]			
No	1.05	0.75	0.79, 1.39
Land owned (acres)	0.91	0.16	0.81, 1.04
Household size	1.002	0.94	0.95, 1.05
Household head education status: literate[2]			
Illiterate	1.47	0.01	1.09, 1.99
Age (years)	0.99	0.03	0.98, 1.00
Employment category: Casual labour[2]			
Regular Salaried	0.68	0.08	0.44, 1.05
Self Employed in Agriculture	0.79	0.20	0.55, 1.13
Self Employed in Non-agriculture	0.68	0.12	0.41, 1.11
MPCE categories[3]: Quintile 1[2]			
Quintile 2	0.90	0.63	0.58, 1.40
Quintile 3	0.87	0.53	0.55, 1.36
Quintile 4	1.00	1.00	0.64, 1.56
Quintile 5	0.67	0.09	0.42, 1.07

[1]*Analysis using logistic regression.*
[2]*Reference category.*
[3]*Monthly per capita expenditure quintiles as a proxy for income quintile.*

For peer review only

Table 5: Cash transfer and Food insecurity for the longitudinal data[1] (N=859)

Food Insecure	Odds Ratio	p value	95% Confidence Interval
Time of assessment: Pre lockdown[2]			
Post Lockdown	4.49	<0.001	3.41, 5.92
Received cash benefits: No[2]			
Yes	1.15	0.43	0.81, 1.61
Time period * Received cash benefits	0.65	0.06	0.42, 1.01
PDS Beneficiary: Yes[2]			
No	1.38	0.41	0.64, 2.98
Land owned (acres)	0.91	0.57	0.67, 1.25
Household size	0.98	0.41	0.93, 1.03
Household head education status: literate[2]			
Illiterate	1.37	0.04	1.02, 1.85
Age (years)	0.99	0.04	0.98, 1.00
Employment category: Casual labour[2]			
Regular Salaried	0.67	0.02	0.48, 0.94
Self Employed in Agriculture	0.70	0.02	0.53, 0.94
Self Employed in Non-agriculture	0.64	0.02	0.43, 0.94
MPCE categories[3]: Quintile 1[2]			
Quintile 2	0.85	0.35	0.60, 1.19
Quintile 3	0.91	0.59	0.64, 1.29
Quintile 4	0.89	0.51	0.62, 1.27
Quintile 5	0.75	0.13	0.51, 1.09
Propensity score	5.93	0.62	0.01, 6794.14

[1]*Analysis using generalized estimation equation model.*
[2]*Reference category.*
[3]*Monthly per capita expenditure quintiles as a proxy for income quintiles.*

For peer review only - http://bmjopen.bmj.com/site/about/guidelines.xhtml

Figure 1: Framework outlay for the data sources used and methodology adopted

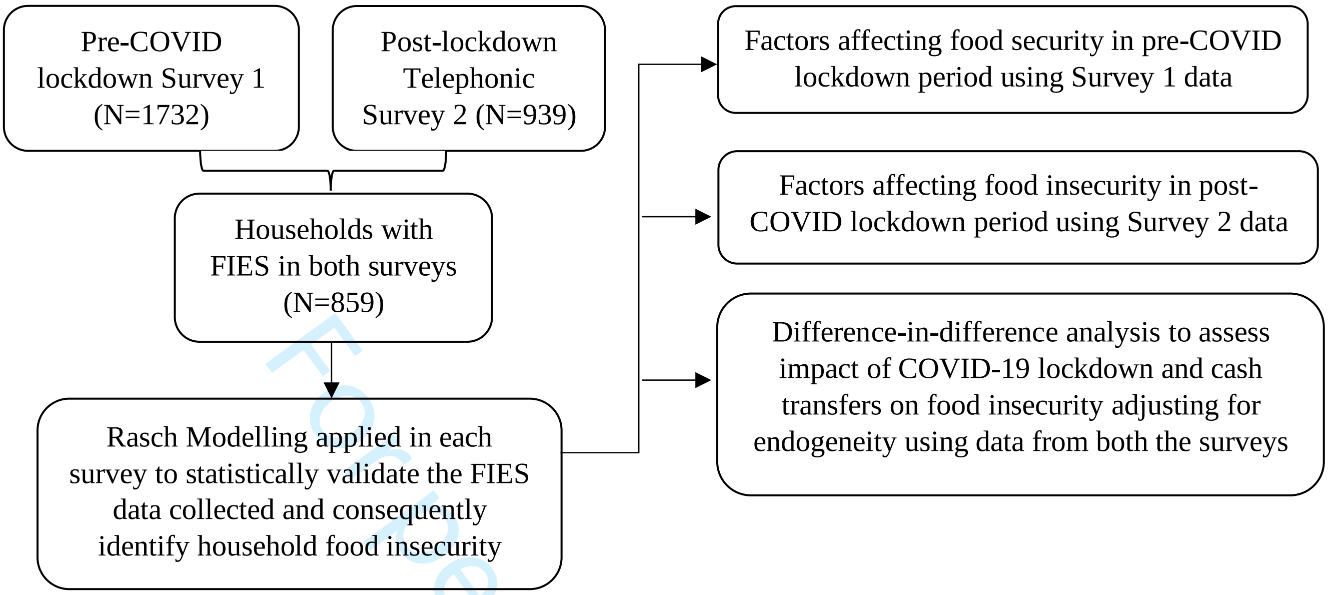

Supplementary Table 1: Household characteristics affecting participation in the Survey 2[1] (N=1713)

Whether the household participated in Survey 2 or not	Odds Ratio	95% Confidence Interval
Food Insecure: No[2]		
Yes	1.05	0.81, 1.35
PDS Beneficiary: Yes[2]		
No	1.02	0.84, 1.24
Land owned (acres)	0.96	0.91, 1.01
Household size	0.98	0.94, 1.02
Household head education status: literate[2]		
Illiterate	1.54	1.26, 1.90
Age (years)	1.00	1.00, 1.00
Positive or no income shock[2,3]		
Negative Income shock	1.09	0.87, 1.38
Employment category: Casual labour[2]		
Regular Salaried	1.01	0.75, 1.36
Self Employed in Agriculture	1.02	0.79, 1.30
Self Employed in Non-agriculture	0.90	0.64, 1.27
MPCE categories[4]: Quintile 1[2]		
Quintile 2	0.82	0.60, 1.11
Quintile 3	0.81	0.59, 1.10
Quintile 4	0.75	0.55, 1.02
Quintile 5	0.86	0.62, 1.18

For peer review only

[1]*Analysis using logistic regression.*
[2]*Reference category.*
[3]*No or positive economic shocks includes found a job, hike in salary and received food and money as gift whereas negative economic shock includes business closures, mass layoffs, price increase of commodities, job loss, wage cuts, loss of remittances, low rate for produce to be sold at market price, indebtedness, and crop failure.*
[4]*Monthly per capita expenditure quintiles as a proxy for income quintile.*

For peer review only

Supplementary Table 2: Household characteristics by cash benefit status in the post lockdown period (N=859)

		Households who did not receive cash benefits (N=497)	Households who received cash benefits (N=362)	p-value
Age(years)		40 [30,58.5]	42 [30,55]	0.79
Household size		7 [5,9]	7 [6,9]	0.078
Land owned (hectares)		0.63 [0.13,2.58]	0.25 [0.08,1.95]	0.016
Household head education	Illiterate	334 (67.2%)	240 (66.3%)	0.78
	Literate	163 (32.8%)	122 (33.7%)	
Household head gender	Male	448 (90.1%)	315 (87.0%)	0.15
	Female	49 (9.9%)	47 (13.0%)	
PDS Beneficiary	Yes	231 (46.5%)	209 (57.7%)	0.001
	No	266 (53.5%)	153 (42.3%)	
Caste category	Forward class	64 (12.9%)	41 (11.3%)	0.79
	Other backward classes	289 (58.1%)	215 (59.4%)	
	Dalit & mahadalits	144 (29.0%)	106 (29.3%)	
Employment category	Casual labourer	117 (23.5%)	91 (25.1%)	0.93
	Regular Salaried	95 (19.1%)	64 (17.7%)	

For peer review only

	Self Employed in Agriculture	229 (46.1%)	166 (45.9%)	
	Self Employed in Non-agriculture	56 (11.3%)	41 (11.3%)	
Household wealth status	Quintile 1	88 (17.7%)	67 (18.5%)	0.66
	Quintile 2	97 (19.5%)	76 (21.0%)	
	Quintile 3	97 (19.5%)	77 (21.3%)	
	Quintile 4	105 (21.1%)	77 (21.3%)	
	Quintile 5	110 (22.1%)	65 (18.0%)	

**Numbers are presented as median (Inter-Quartile range) for continuous measures, and frequency (%) for categorical measures*

For peer review only

STROBE 2007 (v4) Statement—Checklist of items that should be included in reports of *cohort studies*

Section/Topic	Item #	Recommendation	Reported on page #
**Title and abstract**	1	(*a*) Indicate the study's design with a commonly used term in the title or the abstract	1
		(*b*) Provide in the abstract an informative and balanced summary of what was done and what was found	2,3
**Introduction**			
Background/rationale	2	Explain the scientific background and rationale for the investigation being reported	5,6
Objectives	3	State specific objectives, including any prespecified hypotheses	6
**Methods**			
Study design	4	Present key elements of study design early in the paper	6,7
Setting	5	Describe the setting, locations, and relevant dates, including periods of recruitment, exposure, follow-up, and data collection	6,7
Participants	6	(*a*) Give the eligibility criteria, and the sources and methods of selection of participants. Describe methods of follow-up	6,7
		(*b*) For matched studies, give matching criteria and number of exposed and unexposed	N/A
Variables	7	Clearly define all outcomes, exposures, predictors, potential confounders, and effect modifiers. Give diagnostic criteria, if applicable	8-10
Data sources/ measurement	8*	For each variable of interest, give sources of data and details of methods of assessment (measurement). Describe comparability of assessment methods if there is more than one group	6,7
Bias	9	Describe any efforts to address potential sources of bias	9,10
Study size	10	Explain how the study size was arrived at	6,7 (Figure 1)
Quantitative variables	11	Explain how quantitative variables were handled in the analyses. If applicable, describe which groupings were chosen and why	8-10
Statistical methods	12	(*a*) Describe all statistical methods, including those used to control for confounding	8-10 (Figure 1)
		(*b*) Describe any methods used to examine subgroups and interactions	9,10
		(*c*) Explain how missing data were addressed	N/A
		(*d*) If applicable, explain how loss to follow-up was addressed	12 (Supplementary Table 1)
		(*e*) Describe any sensitivity analyses	9,10

For peer review only

**Results**			
Participants	13*	(a) Report numbers of individuals at each stage of study—eg numbers potentially eligible, examined for eligibility, confirmed eligible, included in the study, completing follow-up, and analysed	6-7
		(b) Give reasons for non-participation at each stage	7
		(c) Consider use of a flow diagram	Figure 1
Descriptive data	14*	(a) Give characteristics of study participants (eg demographic, clinical, social) and information on exposures and potential confounders	11 (Table 1), 13 (Supplementary Table 2)
		(b) Indicate number of participants with missing data for each variable of interest	N/A
		(c) Summarise follow-up time (eg, average and total amount)	6
Outcome data	15*	Report numbers of outcome events or summary measures over time	Table 2, Table 5
Main results	16	(*a*) Give unadjusted estimates and, if applicable, confounder-adjusted estimates and their precision (eg, 95% confidence interval). Make clear which confounders were adjusted for and why they were included	11-14 (Table 2-5)
		(*b*) Report category boundaries when continuous variables were categorized	Table 1, Supplementary Table 2
		(*c*) If relevant, consider translating estimates of relative risk into absolute risk for a meaningful time period	N/A
Other analyses	17	Report other analyses done—eg analyses of subgroups and interactions, and sensitivity analyses	13,14
**Discussion**			
Key results	18	Summarise key results with reference to study objectives	14
**Limitations**			
Interpretation	20	Give a cautious overall interpretation of results considering objectives, limitations, multiplicity of analyses, results from similar studies, and other relevant evidence	17
Generalisability	21	Discuss the generalisability (external validity) of the study results	15,16
**Other information**			
Funding	22	Give the source of funding and the role of the funders for the present study and, if applicable, for the original study on which the present article is based	18

*Give information separately for cases and controls in case-control studies and, if applicable, for exposed and unexposed groups in cohort and cross-sectional studies.

For peer review only

Note: An Explanation and Elaboration article discusses each checklist item and gives methodological background and published examples of transparent reporting. The STROBE checklist is best used in conjunction with this article (freely available on the Web sites of PLoS Medicine at http://www.plosmedicine.org/, Annals of Internal Medicine at http://www.annals.org/, and Epidemiology at http://www.epidem.com/). Information on the STROBE Initiative is available at www.strobe-statement.org.

For peer review only
